# On three-dimensional misorientation spaces

**DOI:** 10.1098/rspa.2017.0274

**Published:** 2017-10-25

**Authors:** Robert Krakow, Robbie J. Bennett, Duncan N. Johnstone, Zoja Vukmanovic, Wilberth Solano-Alvarez, Steven J. Lainé, Joshua F. Einsle, Paul A. Midgley, Catherine M. F. Rae, Ralf Hielscher

**Affiliations:** 1 Department of Materials Science and Metallurgy, University of Cambridge, 27 Charles Babbage Road, Cambridge CB3 0FS, UK; 2 Department of Earth Sciences, University of Cambridge, Downing Street, Cambridge CB2 3EQ, UK; 3 Applied Functional Analysis, TU Chemnitz, Germany

**Keywords:** misorientations, orientation relationships, crystallography, diffraction, electron backscatter diffraction, texture

## Abstract

Determining the local orientation of crystals in engineering and geological materials has become routine with the advent of modern crystallographic mapping techniques. These techniques enable many thousands of orientation measurements to be made, directing attention towards how such orientation data are best studied. Here, we provide a guide to the visualization of misorientation data in three-dimensional vector spaces, reduced by crystal symmetry, to reveal crystallographic orientation relationships. Domains for all point group symmetries are presented and an analysis methodology is developed and applied to identify crystallographic relationships, indicated by clusters in the misorientation space, in examples from materials science and geology. This analysis aids the determination of active deformation mechanisms and evaluation of cluster centres and spread enables more accurate description of transformation processes supporting arguments regarding provenance.

## 1. Introduction

Multiphase polycrystalline materials generally contain numerous crystals with different crystal structures. The orientations [1–3] of these crystals and relationships between them [4,5] are important for understanding macroscopic properties [6–8] and microstructural transformation pathways [9–12]. The relationships between crystals are specified by the *misorientation* (rotation) between adjacent crystals as well as the interface boundary where they join [4]. Such a relationship may therefore be parametrized [13] in terms of three misorientation parameters relating the crystal orientations and, when the interface can be approximated as planar, two interface parameters. This leads to a five-parameter description of a crystallographic relationship [4,14,15]. Here, the focus is on the three misorientation parameters [16,17].

Misorientations, between crystals of the same or different phases, often occur repeatedly near to particular values. This is typically the result of special *crystallographic orientation relationships* arising due to the transformation pathway [9,10,12]. Discovering and categorizing these orientation relationships is, therefore, an important element of rationalizing microstructure in materials science. A significant class of orientation relationship results in approximately coincident lattice points when the lattices associated with each crystal are interleaved. These are known as a *coincident site lattice* (CSL) relationships [6,18–21]. The occurrence of exact CSLs in three-dimensional (3D) is quite restricted in non-cubic materials (except for special axial ratios) [4,21–23], but near CSL misorientations often turn out to be significant [24]. Another special class of orientation relationship may be described by a simple rotation of 180° and is known as a *twinning relationship* [6,25,26]. Both of these concepts are invoked in this paper to categorize misorientations.

Orientation relationships can often be well approximated by geometrically simple models expressed as parallelisms between low-index crystallographic planes and directions [27]. Data analysis have traditionally been approached similarly, by attempting to find common poles, e.g. in pole figures. The parallelism description neglects the experimental fact that there is inevitably some spread in measured misorientations. Importantly, this is not only due to errors but also due to local distortions in the microstructure. Furthermore, in some cases, relationships arising due to a phase transformation are necessarily poorly described by low-index parallelisms [28]. Analysis based on the axis and angle of rotation [4,17,29–34], on which this work builds, changes this paradigm.

Crystallographic mapping experiments reveal the phases present and crystal orientations in a spatially resolved manner [35]. Such mapping may be achieved using a number of X-ray [36–38] and electron diffraction [39–42] techniques, which routinely yield many thousands of measurements. Analysing these data to maximize the potential for physical insight remains challenging and has been addressed in the extensively literature [1–3,43]. This paper draws particularly on the insight of Frank [44], who noted that orientation mapping experiments result in ‘a practical need for comprehensible displays of complete orientation statistics. To degrade that information to pole figures, showing the statistics of orientation of crystal planes, and not crystals, is a criminal disregard of significant information’. This triggered interest in 3-vector representations of crystal orientations [4,17,29–34], defining 3D spaces in which to plot the data. The influence of symmetry on the relevant domains of these 3D vector spaces for misorientations has since been studied particularly by Morawiec & Patala [16,17,43]. However, there has been relatively little application of 3D vector spaces in the analysis of experimental data, especially in the context of revealing crystallographic orientation relationships between low-symmetry crystals of different phases.

Computational advances and the growth of open source packages [45–47] now make analysis in 3D orientation space accessible. This paper is intended as a practical guide to 3D orientation spaces with the ultimate goal of revealing crystallographic relationships in multiphase materials. This analysis preserves intrinsic spread in measured values and methods are also developed to explore potential links between particular orientation relationships and spatial occurrence. A review of key concepts in orientation analysis is presented in §2, followed by a discussion of the application of crystallographic symmetry to the 3D orientation spaces in §3. Examples from materials and earth sciences demonstrating application of 3D orientation spaces to glean physical insight are then presented in §4. Details of the calculations performed and important conventions are provided as appendices. All analysis was performed in the matlab toolbox MTEX [45] and scripts are provided as electronic supplementary material at [48].

## 2. Orientations and misorientations

**Figure 1.**
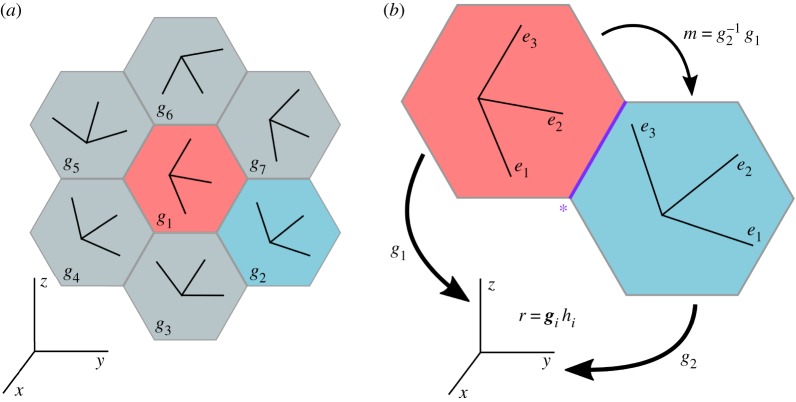
(*a*) Orientations ***g***_*i*_ of crystallographic axes in each structural element (pixel, voxel, or grain) with respect to an external reference frame. (*b*) Orientations, ***g***_*i*_, as transformations from the crystal reference frames, *h*_*i*_, into the specimen reference frame, *r*, and misorientation, ***m***, describing transformation between crystal reference frames across a boundary element (starred).

Crystallographic orientation maps describe, at each position, the crystallographic phase and the directions of the crystallographic basis vectors. Coordinate systems are introduced to specify these directions in terms of a *specimen reference frame*, *r*, and *crystal reference frames*, *h*_*i*_. The local orientation may then be described as a transformation between coordinate systems. Conventionally, the reference frames introduced are orthonormal,^1^ right-handed, and share the same origin to simplify the description. A schematic representation of an orientation map, in these terms, is shown in figure 1*a*.

Orientations are conventionally defined as passive rotations (i.e. tensor quantities are not rotated) that transfer coordinates with respect to a crystal reference system into coordinates with respect to a specimen reference system.^2^ The rotation angle is taken to be positive for a rotation that is counterclockwise when viewed along the corresponding rotation axis towards the origin. An orientation, ***g***, therefore satisfies

2.1 r=gh,

where *r*=(**x**,**y**,**z**) specimen coordinates^3^ and *h*=(**e**_1_,**e**_2_,**e**_3_) crystal coordinates. To manipulate orientations, we note that the corresponding rotations form a non-commutative group [43] implying that inverses exist and rotations are combined associatively.

Misorientations, ***m***, are also passive rotations describing coordinate transformations between crystal reference frames. These two crystals are taken to have orientations ***g***_1_ and ***g***_2_. The misorientation ***m*** between these two crystals is then defined and transforms crystal coordinates *h*_1_ into crystal coordinates *h*_2_, as follows:

2.2 $$\text{m}={\text{g}}_{2}^{-1}{\text{g}}_{1}$$

and

2.3 $$\text{m}h_{1}={\text{g}}_{2}^{-1}{\text{g}}_{1}h_{1}={\text{g}}_{2}^{-1}r=h_{2}.$$

The relationships between coordinate systems, orientations and misorientations are illustrated in figure 1*b*.

### (a) Representations of orientations and misorientations

Orientations and misorientations are described as rotations in 3D space, which can be represented in numerous ways [1–3,43,51,52]. Most common in crystallographic texture analysis is the Euler angle representation, which describes the rotation as three successive rotations about independent coordinate axes through angles, *ϕ*_1_, *Φ*, *ϕ*_2_, in the Bunge (ZXZ) convention [1,2]. This is convenient for series expansion of orientation distribution functions [1], but does not convey efficient computation or intuitive plotting.^4^

Rotations are also described by the group of special orthogonal matrices SO(3). Rotation matrices are useful for transforming tensor quantities, but are not the most computationally efficient representations nor are they convenient for representing orientation distributions. More computationally efficient, but less familiar, is the quaternion representation [43,57], which is a four-parameter description of a rotation reflecting the mathematically natural description of rotations in four dimensions (4D). Quaternions are useful because of an interpretation as forming a 4D algebra with efficient computations. The unit quaternions have a two-to-one relationship with SO(3) and define points on the 3-sphere, $S^{3}$, in 4D Euclidean space. The unit quaternion **q** = (*q*_0_, *q*_1_, *q*_2_, *q*_3_) can also be related to the axis of rotation, described by a unit vector, ***ξ***, via ${\text{q}}_{i}=\sin(\omega /2)\xi_{i}$ for *i*=1,2,3 and the angle of rotation *ω* via $q_{0}=\cos(\omega /2)$.

Rotations may also be represented by the axis and angle of rotation. (Mis)orientation distributions can be expanded in these terms [58,59] and the representation is intuitive. A series of so-called neo-Eulerian mappings have been defined, based on this notion, as 3D vectors formed by scaling a unit vector, ***ξ***, parallel to the axis of rotation by a function of the rotation angle, *ω*, about that axis, *f*(*ω*). The choice of the scaling function, *f*(*ω*), conveys particular properties on the resulting vector space. Five suggestions were made by Frank [44,60], as follows:

*Axis-angle*, **v**=*ω****ξ***: Simple and the angular units ease direct interpretation.^5^
*Rodrigues–Frank*, r=tan⁡(ω/2)ξ: Rectilinear, i.e. rotation about a given axis is a straight line through any point. Some domains are unbounded since the scaling function tends to infinity.
*Conformal*, c=2tan⁡(ω/4)ξ: Equal angle projection of $S^{3}$ onto Euclidean space. The configuration in any small region of the map is geometrically similar to that which it would have, if transferred to any other point in the map.
*Homochoric*, $\text{z}={\left\{\frac{3}{4}(\omega -\sin\omega )\right\}}^{1/3}\xi$: Equivalent of the equal area plane map of a sphere and can be considered an equal volume projection of $S^{3}$ onto Euclidean space. The determinant of the metric tensor is preserved and a random distribution of orientations will have equal probabilities of being found within equal volume elements anywhere in the map [44].^6^
*Quaternion vector*, q=sin⁡(ω/2)ξ: Enclosed within a sphere of unit radius and easily related to the quaternion representation, described above, for computations.

The neo-Eulerian mappings each offer certain advantages. In particular, the rectilinearity of the Rodrigues–Frank representation has made it popular although the unbounded nature of fundamental zones containing rotations of 180° is a practical issue for low symmetry systems. The homochoric representation is attractive for visualization owing to the equal distribution of randomly distributed points [17]. Analysis principles developed in this work apply equally well to all neo-Eulerian mappings, which have all been made available in the open-source MTEX toolbox so that readers may make the most appropriate choice for their needs. Here, we apply the axis-angle parametrization because it is sufficient to illustrate the important principles, bounded in low-symmetry cases, and the magnitude of the vector is directly the misorientation angle which simplifies at-a-glance interpretation.

## 3. Fundamental zones and crystal symmetry

Crystal symmetry implies that some (mis)orientations are physically indistinguishable. However, equivalent (mis)orientations will be represented by different points in the 3D vector space when expressed as neo-Eulerian vectors. It is only necessary to use a region of the vector space containing each physically distinct (mis)orientation precisely once. Such a region is known as a *fundamental zone* [4,33,44] or an *asymmetric domain* [16]. In this section, a consistent definition for the fundamental zone is set out and fundamental zones are tabulated for all crystal symmetries.

**Figure 2.**
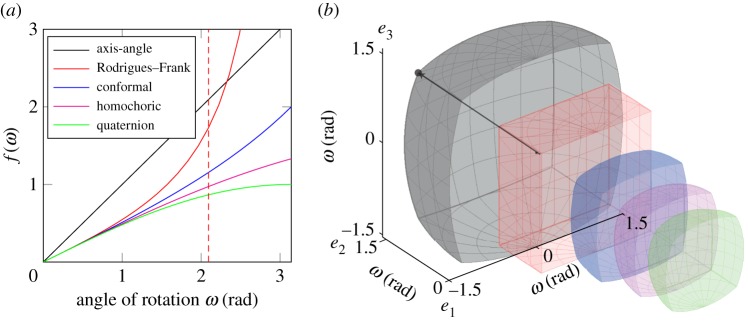
(*a*) The scaling function for five neo-Eulerian orientation mappings as a function of the rotational angle. The maximum angle for 222 symmetry is 2*π*/3 radians (120°) in [111] direction (indicated by red dotted lines). (*b*) Sectioned fundamental zone for 222 symmetry in each of the five neo-Eulerian mappings, illustrating differences in geometry.

Fundamental zones have previously been specified by a number of authors based on Rodrigues–Frank parameters [4,16,33]. Here, the calculation was instead achieved following a construction based on quaternion geometry, as detailed in appendix A. The fundamental zone may then be transformed into any of the neo-Eulerian representations. These representations differ geometrically, as discussed in §2. The scaling functions, *f*(*ω*), shown in figure 2*a*, are all approximately linear up to approximately 1 radian and then diverge. The bounding surfaces of the fundamental zone are curved in all cases except Rodrigues–Frank, see figure 2*b*, reflecting the aforementioned rectilinearity.

The particular fundamental zone obtained depends on the alignment of axes and the order in which symmetry operators are combined for misorientations. The standard settings, consistent with the International Tables for Crystallography, are followed here and these conventions are detailed in appendix B. The importance of these alignments cannot be understated since inconsistent adoption of conventions for these alignments can cause much confusion in applying the methods described here.

### (a) Symmetry equivalence of (mis)orientations

Calculation of the fundamental zone is based on selecting, from symmetry equivalent points, the point closest to the origin (smallest angle of rotation) and rejecting more remote points. When multiple equivalent points have the same distance to the origin, a constraint is placed on the direction of the axis of rotation. To express this mathematically, it is noted that crystal coordinates are subject to symmetry described by the group of symmetry operators, ***s***, comprising the crystallographic point group *S*. A symmetry operation applied to the crystal coordinates produces a physically identical configuration and therefore the crystal coordinates *h* can be identified with a set ***s****h* (*s*∈*S*) of symmetrically equivalent crystal coordinates.^7^ Considering equation (2.1) yields the following expression for symmetrically equivalent orientations,

3.1 g=gs,s∈S.

Misorientations are subject to the symmetry operations of crystallographic point groups, *S*_1_ and *S*_2_, associated with the crystal coordinate systems, *h*_1_ and *h*_2_, which are related by the misorientation, ***m***. Considering the effect of symmetry on each crystal coordinate system, as described above, and using equation (2.3), the following expression for symmetrically equivalent misorientations is obtained.

3.2 $$\text{m}=s_{2}\text{m}s_{1},s_{1}\in S_{1},s_{2}\in S_{2}.$$

Unique selection of a misorientation (referred to as the *disorientation)* to represent all symmetrically equivalent misorientations^8^ requires a constraint on both the angle of rotation and axis of rotation because several symmetrically equivalent misorientations may have the same rotational angle.^9^ Here, the misorientation with the smallest angle of rotation (known as the disorientation angle) and an axis of rotation within the inverse pole figure (IPF) sector corresponding to the point group of common symmetries, *S*_*C*_=*S*_1_∩*S*_2_, is chosen.

### (b) Relating symmetry to fundamental zones

Construction of the fundamental zone may be understood by considering that the symmetry operators map the reference (mis)orientation or identity, which is at the origin of the 3D vector space, to a set of *identity equivalent points* throughout the vector space following equations (3.1) and (3.2). The fundamental zone then comprises the set of points that are closer to the identity at the origin than any of the other identity equivalent points. Indeed, this is precisely the notion used by Morawiec [16] and in this work (appendix A) to compute the fundamental zone. This view of the construction is similar to that commonly used to describe the Brillouin Zone in reciprocal space and helps to rationalize the observed shapes of the fundamental zone, as below.

Fundamental zones for orientations involve only one set of symmetry operators according to equation (3.1). This is also equivalent to the formation of a misorientation fundamental zone when *S*_2_=1. A two-fold symmetry axis produces an identity equivalent point at a position 180° from the origin along the symmetry axis. Points up to 90° from the origin along this axis are clearly closer to the identity at the origin than to the identity equivalent point and therefore the boundary of the fundamental zone is at 90° along this axis. This idea extends easily to other rotational symmetry operators with triads, tetrads and hexads each producing identity equivalent points at 120°, 90° and 60° along the respective symmetry axis. Visualizing the curvature of the bounding surfaces of the fundamental zone is more difficult and depends on the particular representation chosen. The principle is to construct the surface corresponding to rotation about axes orthogonal to the symmetry axis and passing through the easily defined bounding point on the symmetry axis. The inner envelope of these surfaces will then define the fundamental zone.

**Figure 3.**
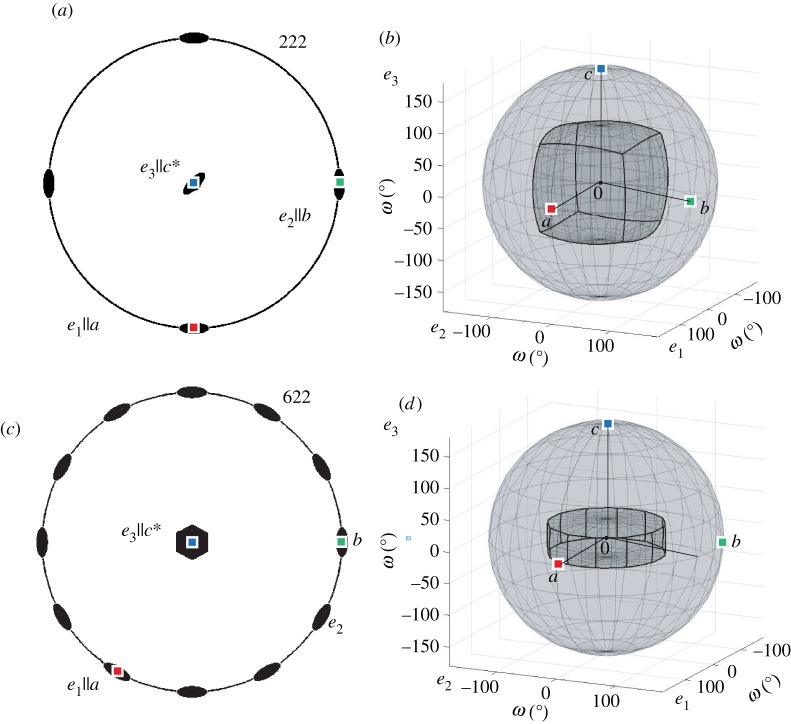
(*a*,*c*) Stereographic projections of symmetry elements and corresponding symmetry reduced *fundamental zones* (*b*,*d*) in axis-angle space for: (*a*,*b*) orientations of crystals with point group *222* (*c*,*d*) orientations of crystals with point group *622*.

Considering the *222* point group, the diad operators constrain the domain to ±90° along the coordinate axes producing a convex cube, as shown in figure 3*b*. For the *622* point group, the hexad operator constrains the domain to ±30° along ***e***_3_ and the diad operators constrain the domain to ±90° along each of the corresponding axes, as shown in figure 3*d*. It is, therefore, reasonably intuitive to deduce the qualitative shape of the fundamental zone for orientations.

**Figure 4.**
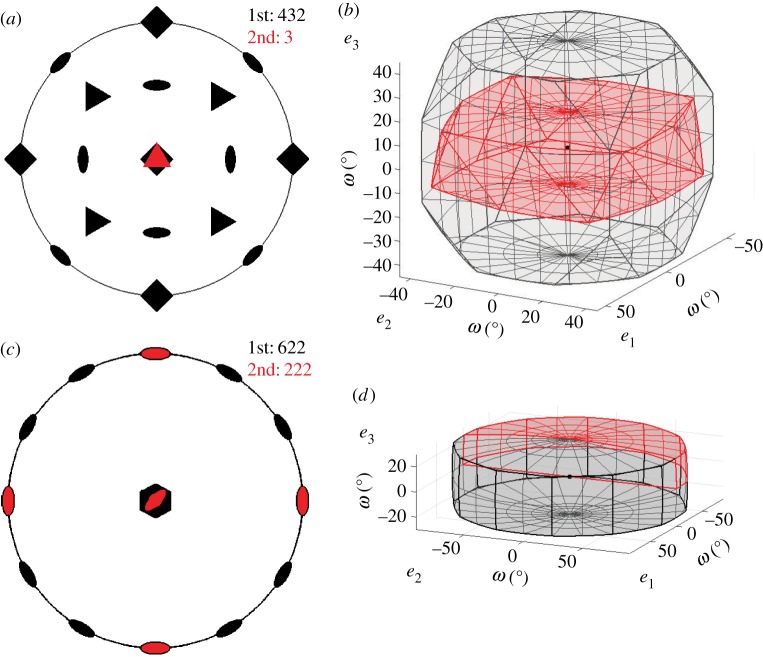
Stereographic projections (*a*,*c*) of symmetry elements and corresponding symmetry reduced *fundamental zones* of axis-angle space (*b*,*d*) for: (*a*,*b*) misorientations of crystals with point group (PG) *432* and combination of PGs *432*-*3* (*c*,*d*) misorientations of crystals with PG *622* and combination of *622*-*222*.

Fundamental zones for misorientations when both crystals contribute symmetry operators are formed in two different ways depending on whether the symmetry groups contain common elements as discussed in more detail by Morawiec [16]. When the two symmetry groups do not contain any common elements, then there are no symmetry equivalent misorientations with the same misorientation angle and the fundamental zone is again constructed by selecting the misorientation closest to the origin. This is the case for the *432*-*3* fundamental zone shown in figure 4*a*,*b*. If the symmetry groups do contain common elements, then there will be symmetry equivalent misorientations with the same angle of rotation and the restriction on the misorientation axis discussed above is required. This is the case for the *622*-*222* fundamental zone shown in figure 4*c*,*d*. All of the symmetrically equivalent misorientations with the same misorientation angle lie within the higher symmetry *622* fundamental zone. Each diad from the *222* point group then effectively excludes half of the space. However, a pair of diads implies the third and therefore the fundamental zone is $\frac{1}{4}$ the original domain rather than $\frac{1}{8}$. The particular segments defining the fundamental zone correspond to the defined IPF segment.

The segmentation seen in figure 4*d* leads to a notion of *domain geometries* where the fundamental zone is either (i) one of the domain geometries (e.g. figure 3 or 4*b*) or (ii) a segment of it, produced by cutting with planes often parallel to **e_1_**, **e_2_** or **e_3_**, as in figure 4*d*. This makes it tractable to discuss fundamental zones for all crystal combinations, as follows.

### (c) Fundamental zones for all crystals

Crystals possess point group symmetry described by one of the 32 crystallographic point groups. Eleven of these point groups are *proper point groups* and only contain symmetry operations that are proper rotations. The remaining crystallographic point groups contain improper symmetry operations that involve inversion. Treatment of improper operations has varied somewhat between authors. Here, we adopt the convention in which the crystals may only be related by a proper rotation operation and therefore only the proper point groups must be considered. A correspondence table between the Laue class of a crystal and the appropriate proper point group for definition of the fundamental zone is provided in appendix C to inform the correct choice.

**Figure 5.**
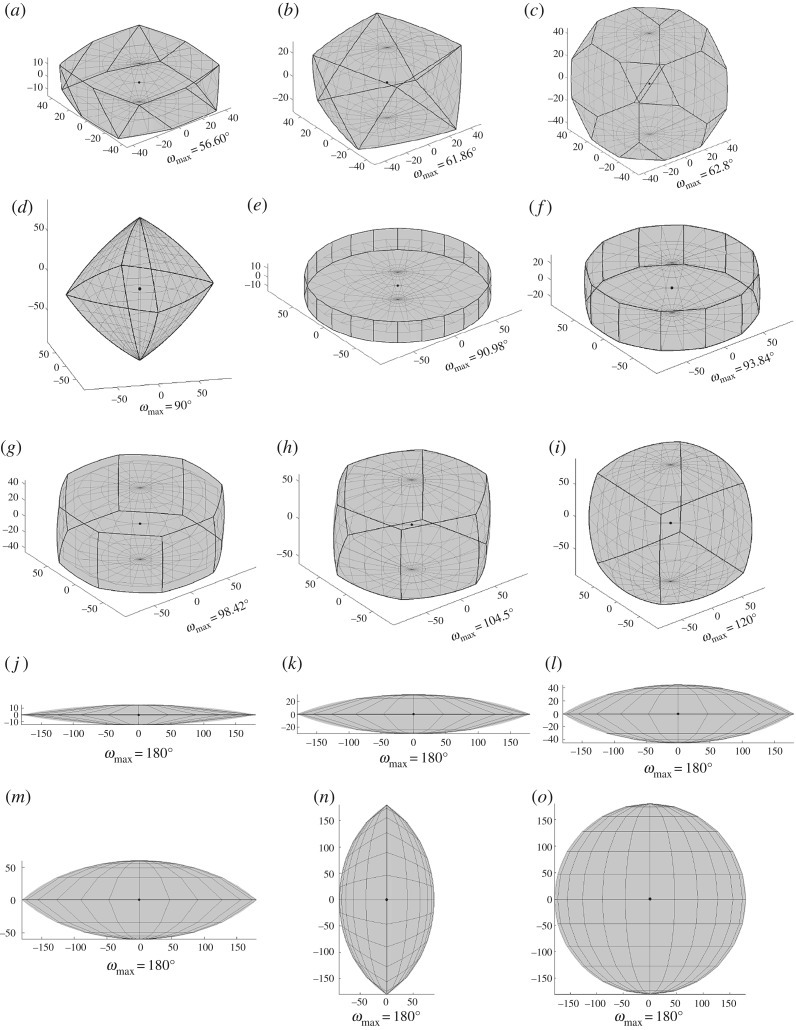
Domain geometries in axis-angle representation with the reference misorientation at the origin (marked with a point). Combinations of point group symmetries leading to fundamental zones that are one of these domain geometries or a section of it are provided in table 1.

Fundamental zones for all proper point group combinations were computed and it was found that 15 distinct *domain geometries* occur, as shown in figure 5*a*–*o*, for increasing maximum angle of rotation. The correspondence between fundamental zones for misorientations and these domain geometries is summarized in table 1.

**Table 1.** Fundamental zones for misorientations expressed as sections of the domain geometries shown in figure 5 for all combinations of proper point group symmetry operators, which are listed in Hermann–Mauguin notation [6]. The self-symmetry combinations (highlighted) do not include so-called grain exchange symmetry, which would halve the domain space.

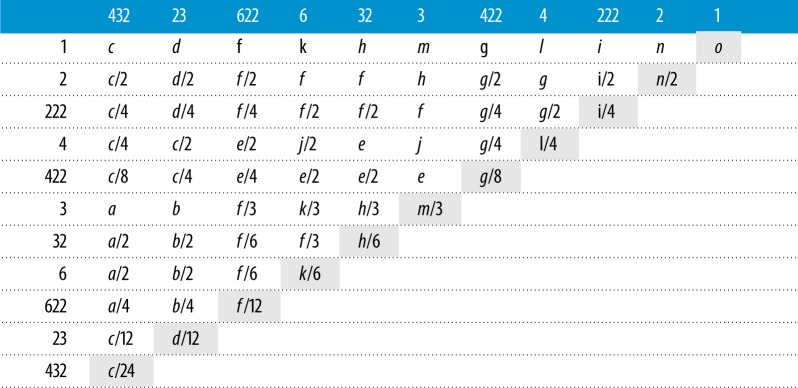

Of the 15 domain geometries, 11 (all excluding figure 5*a*,*b*,*e*,*j*) correspond directly to the fundamental zones for orientations and, equivalently, misorientations with *S*_2_=1. The effect of rotational symmetry operators in truncating the orientation space perpendicular to the axis of rotation can be identified in each case, as discussed in the previous section. This is particularly clear for the lenticular fundamental zones (figure 5*o*,*n*,*m*,*l*,*k*) which are formed with the application of: no symmetry, a diad axis, a triad axis, a tetrad axis, and a hexad axis parallel to the short axis of the fundamental zone. The maximum misorientation angle (from the centre) along the axis parallel to this symmetry axis is then limited to 180°, 90°, 60°, 45° and 30°, respectively. Conventional crystallographic settings, with the monoclinic diad parallel to e_2_ result in domain *n* being truncated along the e_2_ axis, whereas others are truncated along e_3_.

Fundamental zones for misorientations combining proper point groups that do not possess common symmetry elements define new domain geometries centred around the origin, as described above. These are the domain geometries in figure 5*a*,*b*,*e*,*j*. In all other cases, the fundamental zone is a segment of one of these 15 domain geometries with a volume indicated by the associated fraction in table 1. The fundamental zones calculated in this work agree with those reported for various proper point group combinations [4,16,17,30,62,63] when differences in axis alignment are accounted for. Here, conventions for axis alignment and the order of symmetry operations have been clearly set out (§3 and appendix B) and the fundamental zone has been reduced to the minimal disorientation space in all cases. The explicit statement of consistent ‘standard’ conventions is practically significant when comparing multiple datasets.

An additional point applies to grain boundary misorientations where the two crystals are of the same phase, i.e. *S*_1_=*S*_2_. In this case, it is not possible to distinguish misorientation ***m*** between grain A and grain B from the inverse misorientation ***m***^−1^ between grain B and grain A. This effectively introduces an additional symmetry, known as *grain exchange symmetry* [16,17,64,65] which halves the fundamental zone.

### (d) Boundaries of the fundamental zone

The fundamental zone contains each symmetrically equivalent misorientation once. In the interior of the fundamental zone, the vector space behaves approximately like the ordinary 3D Euclidean space. In particular, misorientations clustering randomly around a fixed orientation relationship appear as one cloud. However, when plotting the fundamental zone as a bounded domain in 3D space, it is important to consider a misorientation lying on the boundary and the appearance when a cluster of misorientations crosses the boundary.

**Figure 6.**
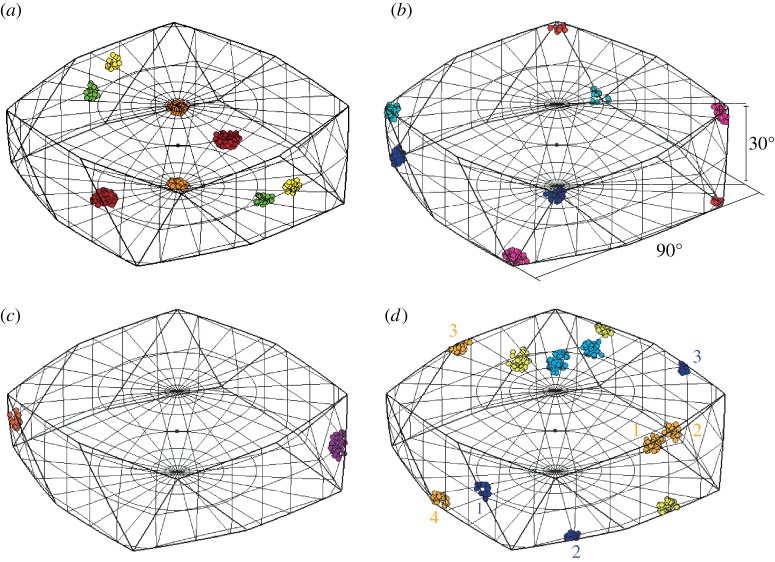
Properties of misorientation clusters situated at the bounding surface of the cubic ($m\bar{3}m$) to trigonal ($\bar{3}$) fundamental zone (domain a). This is illustrated by colouring similar misorientations with equal colours for each image separately. (*a*) Clusters reappearing at an opposite boundary face, either directly opposite or opposite and rotated; (*b*) clusters at a corner reappearing at another corner; (*c*) clusters that do not reappear in a different position; (*d*) clusters at edges and triple points can split into more than two clusters.

A misorientation lying on the boundary of the fundamental zone is equivalent to another misorientation on the boundary with the same rotational angle [44]. A cluster crossing the boundary will, therefore, reappear at this symmetry equivalent boundary point. This is similar, in principle, to crossing the boundary of a Brillouin zone in reciprocal space. However, the topology of the space is more complicated for orientations and rotations are also involved. The most common situation is that a cluster reappears just at the opposite face, but rotated about the face centre, as shown in figure 6*a* for the *432-3* fundamental zone. A cluster crossing the fundamental zone boundary at a corner typically reappears at a different corner, as shown in figure 6*b*. At some edges (and corners in other fundamental zones), the cluster may re-enter at an immediately adjacent point, as shown in figure 6*c*. Finally, less intuitive splitting can occur including: re-entry through a nearby face and splitting into more than two clusters, as shown in figure 6*d*.

In cases where clusters cross the fundamental zone boundary, visualization may be aided significantly by colouring symmetry-related clusters based on misorientation from a suspected orientation relationship or a cluster centre, as shown in figure 6. It can also be helpful to inspect the symmetrized dataset prior to reducing the data to the fundamental zone as it is the application of symmetry that results in the splitting. Finally, it may be advantageous to move away from ‘standard’ conventions for the fundamental zone definition to better reflect the data, as follows.

### (e) Alternative axis alignments

**Figure 7.**
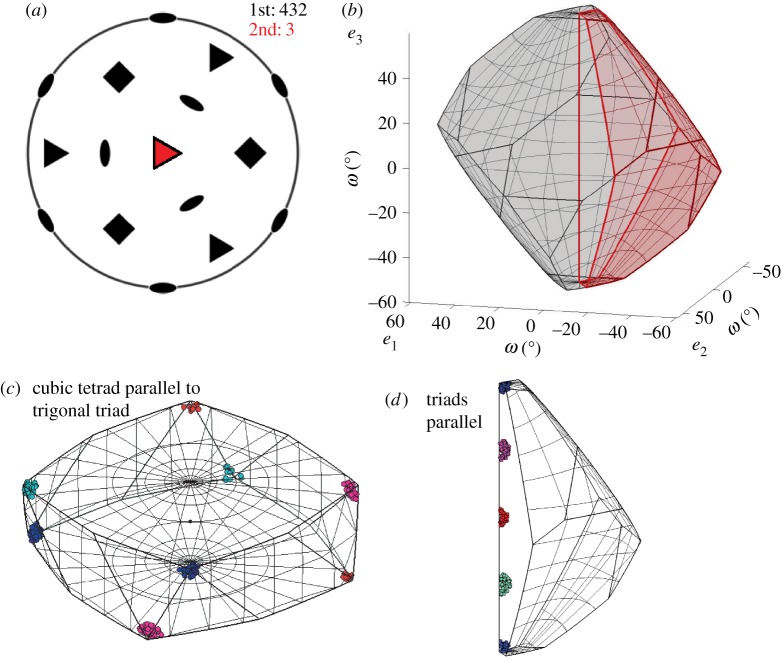
(*a*) Stereographic projection of symmetry elements for cubic ($m\bar{3}m$) and trigonal ($\bar{3}$) point groups with triad axes parallel to e_3_. (*b*) Corresponding symmetry reduced *fundamental zones* (*c*), clusters at vertices, (*d*) clusters along the triad axis (e_3_-axis). Set-up highlights that the four misorientations are rotations about e_3_-axis (c.f. §4).

Adopting ‘standard’ conventions for the alignment of coordinate and symmetry axes has the advantage of familiarity with a misorientation space and enables direct comparison between datasets. However, alternative axis alignments may simplify the interpretation of data by reflecting underlying crystallographic symmetry [16]. For example, the cubic–trigonal fundamental zone for misorientations has the trigonal triad axis and the cubic tetrad axis aligned following the standard conventions, as shown in figure 4*a*,*b*. Crystallographically, it may make sense to align the triad axes of the trigonal and cubic systems, as illustrated in figure 7*a*,*b*. This alternative alignment leads to a fundamental zone of geometry *c*/3, whereas the standard alignment leads to geometry *a*. The alignment of triad axes is particularly advantageous if these symmetry axes are aligned in important crystallographic orientation relationships. In this alternative alignment, misorientations about the triad axis are situated along the e_3_ axis rather than at vertices in the standard fundamental zone, as shown in figure 7*c*,*d*.

## 4. Orientation relationships revealed in misorientation space

Crystallographic orientation relationships, arising throughout materials and earth sciences, may be visualized in 3D misorientation spaces. Here, the utility of this approach is illustrated using examples selected to incorporate important materials systems and a range of crystal symmetries. For each example, a minimal introduction to the key microstructural features is provided and the data, as well as extended analysis scripts, have been made available [48]. The orientation mapping data were obtained via electron backscatter diffraction (EBSD) using conventional Hough-transform-based analysis, which conveys a (mis)orientation measurement precision on the order of approximately 1° [66]. Orientation relationships are assessed by calculating misorientations between all combinations of phases present and plotting them in the respective fundamental zone. When particular crystallographic orientation relationships are preferred, numerous disorientations will be observed to occur close to a particular point in the fundamental zone. The mean of such a cluster of disorientations can then be determined and this is achieved here using the iterative procedure described by Bachmann *et al.* [67]. Typically, such disorientation clusters contain greater than 100 discrete measurements and the mean can, therefore, be determined with a precision of approximately 0.1°. Often cluster centers will be close to a disorientation corresponding to the parallelism of low Miller index planes and directions, which relates the analysis back to more traditional approaches and makes it easier to visualize approximate atomic structures. Finally, boundary segments associated with disorientations in selected clusters may be plotted to assess the microstructural distribution of particular disorientations.

### (a) Orientation relationships in nanostructured bainitic steel

**Figure 8.**
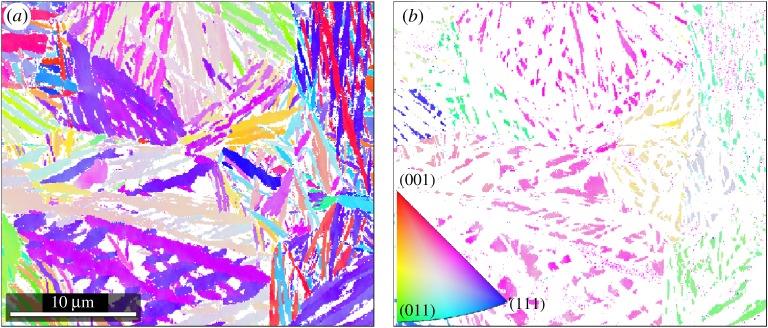
Nanostructured bainite [69], IPF colour code with regard to *z*-axis for both maps in the inlay. (*a*) Orientation map of ferrite laths in IPF colours. Several orientations of laths occur within a prior austenite grain. (*b*) Orientation map of the retained austenite phase in IPF colours.

Nanostructured bainitic steels typically contain key alloying additions of Si, Mn and Ni and the mechanical properties obtained offer a balance of strength and toughness attractive in many applications. The sample studied here was developed for rolling contact fatigue properties [68] as a bearing steel and is described further by Solano *et al.* [69]. The microstructure comprises a dense distribution of ferrite, *α*-Fe, (*m*$\bar{3}$*m*, space group 229) laths in prior austenite, *γ*-Fe, (*m*$\bar{3}$*m*, space group 225) grains, as shown in figure 8. Iron carbides were not observed [69]. Ferrite laths account for approximately 84% of indexed pixels, whereas retained austenite makes up approximately 16%. This is sufficient to recognize the prior austenite grains in figure 8*b*. At least three variants (colours) of ferrite laths are present in each austenite grain, indicating different transformation directions although the full number is not evident from a single IPF coloured map.

Multiple ferrite orientations occur within each prior austenite grain because the transformation from austenite to ferrite can start at any of the equivalent {111}_*γ*_ planes and occur in any of three 〈110〉_*γ*_ directions in each of those planes. Typically, the most densely packed atomic planes in each phase ({110}_*α*_ and {111}_*γ*_) remain nearly parallel [70]. However, the existence of an invariant line, necessary for a glissile interface, requires a shear transformation and a rigid body rotation. The invariant line requirement implies that the habit plane and the resulting orientation relationship must have irrational Miller indices. Several orientation relationships are listed in table 2, but are only approximate. Therefore using 3D misorientation space, the distinction between experimental data and these approximations becomes clear.

**Table 2.** List of common orientation relationships, ferrite–ferrite boundary misorientations and CSL boundaries (for combinations of fcc-fcc and bcc-bcc) used in the analysis [28,71–73].

orientation relationship	definition of misorientation
Kurdjumov–Sachs (KS)	${\left(111\right)}_{\gamma }∥{\left(011\right)}_{\alpha },{\left[\bar{1}01\right]}_{\gamma }∥{\left[\bar{1}\bar{1}1\right]}_{\alpha }$
Nishiyama–Wassermann (NW)	${\left(111\right)}_{\gamma }∥{\left(011\right)}_{\alpha },{\left[11\bar{2}\right]}_{\gamma }∥{\left[0\bar{1}1\right]}_{\alpha }$
Pitsch (P)	${\left(010\right)}_{\gamma }∥{\left(101\right)}_{\alpha },{\left[101\right]}_{\gamma }∥{\left[\bar{1}11\right]}_{\alpha }$
cluster centre	44.2°*about* [−0.1917,0.0996,0.9764]_*γ*_
NW–NW boundary, same {111}_*γ*_	60°*about* [011]
KS–NW boundary, same {111}_*γ*_	54.7°*about* [011]
CSL3 and CSL7*	60° and 38.2°*about* [111]
CSL5*	36.9°*about* [001]
CSL9 and CSL11*	38.9° and 50.5°*about* [011]

**Figure 9.**
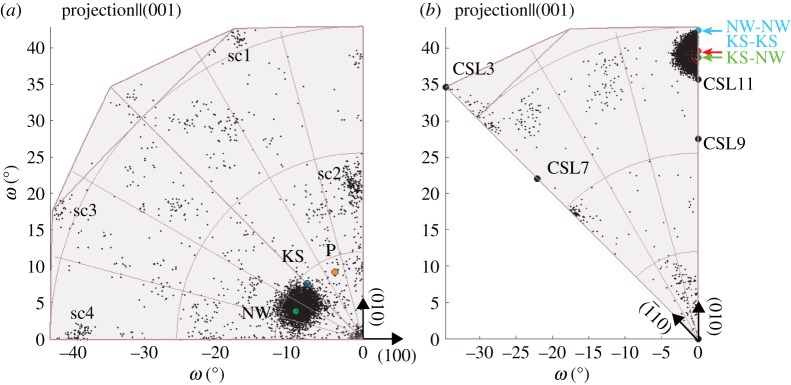
Misorientation distributions of nanostructured bainite in axis-angle space (*a*) Austenite–ferrite distribution with cluster situated between NW and KS (*b*) Ferrite–ferrite distribution with cluster above CSL11. Orientation relationships and CSLs are annotated (table 2).

Austenite-ferrite misorientations in this sample are clustered between the Nishiyama–Wassermann (NW) and Kurdjumov–Sachs (KS) orientation relationships, as shown in figure 9*a*. The radius of the cluster is 2.7°, indicating a misorientation spread greater than the precision of the experiment. This is consistent with other studies on carbide-free bainite [28]. For upper bainite, distributions between KS and NW have been observed, while for lower bainite, a wider misorientation distribution has been observed [74]. The centre of this austenite-ferrite cluster was determined to be 44.2° approximately [−0.1917, 0.0996, 0.9764], which is 1.9° away from the NW orientation relationship that is the closest low-index parallelism. However, the cluster centre provides a more accurate description of the transformation process than any of the established ORs, which is evidenced by the fact that 62.0% of all misorientations are situated at a 2° distance from that centre compared to 28.2% and 2.6% for NW and KS, respectively.^10^ Smaller clusters (sc1–sc4) were found to belong to isolated pixels not clearly associated with the transformation.

Ferrite–ferrite misorientations are cluster centred around a misorientation of 56° about the [011] axis (red arrow). This cluster appears primarily to be a direct consequence of the austenite to ferrite transformation process, described above. This leads to particular misorientations between ferrite laths within any given prior austenite grain and it is clear by the inspection of the orientation maps that the majority of ferrite–ferrite boundaries are situated within prior austenite grains and so dominate the statistics. Flower *et al.* [73] calculated the expected ferrite–ferrite misorientations between two NW-type laths formed in the same parent austenite grain to be 60° about [011] (same for two KS laths) and 54.7° about [011] for a KS lath in contact with a NW variant. These are indicated by blue and green arrows, in figure 9*b*. Like the austenite–ferrite misorientations then the observed ferrite–ferrite misorientation cluster at 56° about the [011] axis is therefore most accurately described with a single cluster of austenite–ferrite misorientations between KS and NW. It is neither a consequence of identical boundary types (60°) nor strongly dissimilar boundaries (54.7°) only. Further, it is noteworthy that he clustering of ferrite–ferrite misorientations may provide a clear criterion for the reconstruction of prior austenite grains.

This case study demonstrates the utility of 3D misorientation spaces in providing a description of the austenite–ferrite transformation that is more consistent with the physical nature of the transformation, which would lead to representation in terms of irrational Miller indices. The description of the orientation relationship as being around the cluster centre is also more accurate in the sense that 61.9% of all misorientations are situated within a distance of 2° of the determined centre, whereas only 28.2% are situated around NW and 2.6% are situated around KS. Finally, this example illustrates the application of grain exchange symmetry for misorientations between the same phase crystals, which halves the domain space for ferrite–ferrite misorientations compared to austenite–ferrite misorientations (see appendix B).

### (b) Deformation twinning in titanium during high strain rates

Deformation twinning occurs in hexagonal close packed (h.c.p.) titanium (6/*mmm*, space group 194) as is typical in h.c.p. metals in order to satisfy the criterion of five independent slip systems for general plastic deformation [75]. Four independent systems are provided by 〈*a*〉 type slip within basal {0001}, prismatic $\left\{1\bar{1}00\right\}$ or pyramidal $\left\{1\bar{1}01\right\}$ planes. Slip activation and hence strain accommodation parallel to the *c*-axis, however, must occur via 〈*c*+*a*〉 type slip along the pyramidal planes, $\left\{1\bar{1}01\right\}$ and $\left\{11\bar{2}2\right\}$, in the $\left\langle 11\bar{2}3\right\rangle$ directions. The critically resolved shear stress for 〈*c*+*a*〉 slip to occur is known to be far greater than for the four other slip systems [76] and therefore deformation parallel to the *c*-axis is largely accommodated by deformation twinning. The deformation twinning modes considered active in h.c.p. titanium are listed in table 3.

**Table 3.** Deformation twins possible in h.c.p. titanium [77]. The misorientation is dependent upon the *c/a* ratio (taken to be 1.588). The mode of twinning refers to whether the *c*-axis of the parent grain is under compression (C) or tension (T). N-CSL values were taken from [20] and compared with calculations using the same algorithm.

twinning plane	misorientation axis/angle	mode	nearest n-CSL
$\left\{11\bar{2}1\right\}$	[10$\bar{1}$0] 34.9°	T	n-CSL11a
$\left\{11\bar{2}2\right\}$	[10$\bar{1}$0] 64.4°	C	n-CSL7a
$\left\{11\bar{2}4\right\}$	[10$\bar{1}$0] 76.9°	C	n-CSL13a
$\left\{10\bar{1}2\right\}$	[11$\bar{2}$0] 85.0°	T	n-CSL11b
$\left\{10\bar{1}1\right\}$	[11$\bar{2}$0] 57.2°	C	n-CSL13b

According to the calculations by Bonnet *et al.* [20], there are near CSL misorientations which have been used to describe twinning misorientations in Ti [78]. It should be noted that these are not strain free in h.c.p. titanium, so have been labelled as near coincident (n-CSL). In addition, since several n-CSL geometries exist for a given strain criterion (such as that chosen in [20]), those nearest to the twinning misorientation are listed in table 3.

**Figure 10.**
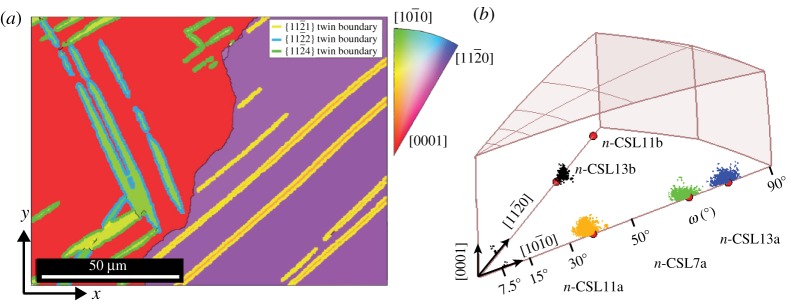
(*a*) Twinned microstructure of ballistically tested pure Ti, adapted from [77]. The loading axis is parallel to the *x*-direction. IPF colours are with respect to the *x*-axis. (*b*) Fundamental zone (*f*/24, cf. table 1) for 6/*mmm* misorientations. Clusters of grain boundary misorientations corresponding to $\left\{11\bar{2}1\right\}$, $\left\{11\bar{2}2\right\}$ and $\left\{11\bar{2}4\right\}$ twin boundaries are colourized and highlighted in the microstructure. The grain boundary misorientation is close to the $\left\{10\bar{1}1\right\}$ twinning OR and is coloured black.

Deformation by twinning can be promoted at high strain rates, because it occurs at a faster rate than slip. Here, room temperature ballistic testing at a strain rate of approximately 10^3^ *s*^−1^ was applied to commercial purity titanium as discussed elsewhere [77], producing a twinned microstructure containing multiple twin variants. In figure 10, the resulting twinned microstructure is shown with three twin types identified and highlighted spatially (figure 10*a*) and in the fundamental zone (figure 10*b*) for the combination of 6/*mmm* with 6/*mmm* symmetry. Grain exchange symmetry halves the fundamental zone for misorientations between crystallites of the same phase (cf. table 1). Three misorientation clusters along the [10$\bar{1}$0] axis corresponding to $\left\{11\bar{2}1\right\}$, $\left\{11\bar{2}2\right\}$ and $\left\{11\bar{2}4\right\}$ twin types are indicated. A cluster of misorientations close to that of the $\left\{10\bar{1}1\right\}$ twin type can also be seen, although this was not a deformation twin, but instead the grain boundary misorientations corresponding to the black line in the centre of figure 10*a*. Indeed, it appears that the $\left\{10\bar{1}1\right\}$ twin mode may not be active under high strain rate deformation in h.c.p. titanium [77]. The occurrence of $\left\{10\bar{1}2\right\}$ twin boundaries was observed in a ballistically tested sample [78], but are not present in the dataset analysed for this study [77].

Spatially (figure 10*a*) it can be seen that $\left\{11\bar{2}1\right\}$ twins form only in the right-hand grain, whereas $\left\{11\bar{2}2\right\}$ and $\left\{11\bar{2}4\right\}$ twins are present in the left grain. This can be explained by the alignment of the parent grain relative to the compressive loading axis. Since the inclination of the *c*-axis to the *x*-direction (loading direction) in figure 10*a* is high (approx. 60°) in the right-hand grain, tensile twinning is the dominant mechanism, hence $\left\{11\bar{2}1\right\}$ twins are formed. In the left-hand grain, compressive twins are present as the *c*-axis is aligned with the loading direction. It is clear that being able to correlate the twinning misorientations back to the microstructure by identifying clusters is valuable in this context. The centres of the orange, green and blue clusters in figure 10*b* have been determined. The respective misorientations were found to differ from the values in table 3 more than previously reported values measured analysing individual selected area diffraction (SAD) patterns [79] based on transmission electron microscopy. These are listed in table 4.

**Table 4.** Cluster centres for the three deformation twinning modes identified in figure 10. The distance from the calculated twinning misorientation is defined as *Δ*M, which is compared to values obtained using SAD by Song *et al.* [79] for a *c/a* ratio of 1.589. The spread around each twin relationship can also be considered individually and is approximately 4.5° on average.

twinning plane	cluster centre	*Δ*M	2D *Δ*M (SAD) [79]
$\left\{11\bar{2}1\right\}$	35.0°	1.1°	0.5°
$\left\{11\bar{2}2\right\}$	64.4°	1.0°	0.5°
$\left\{11\bar{2}4\right\}$	76.9°	0.6°	—
$\left\{10\bar{1}2\right\}$	—	—	0.5°

The misorientation error *Δ*M between cluster centres and calculated twinning misorientations shown in table 4 is larger than that previously reported [79] and is most likely due to the limitations of SAD with the beam parallel to the twinning misorientation axis [1$\bar{1}$00], where only in-plane rotations of the diffraction pattern can be measured to obtain a misorientation angle.

This case study demonstrates the easy identification of twinning modes in h.c.p. titanium. Using the appropriate fundamental zone, the twinning misorientations can be used to highlight specific clusters of misorientations in axis-angle space and see where those clusters occur within the microstructure. The clusters themselves are found to be centred about twinning misorientations that differ from calculated values (0.6–1.1°), an observation that was previously calculated using planar rotations in SAD patterns. The fact that *Δ*M can be determined in 3D for each twinning mode demonstrates the superiority of statistical analysis using a 3D vector space.

### (c) Precipitate behaviour in an advanced nickel-superalloy

Nickel-base superalloys have risen to prominence in the field of aerospace materials, when high temperature capability is of primary importance [80]. Recently, the ATI718Plus${\;}^{®}$ alloy has been developed for static and rotating applications [81]. In ATI718Plus, hexagonal (6/*mmm*, space group 194) *η*-phase precipitates are formed within the cubic (*m*$\bar{3}$*m*, space group 225) *γ*-Ni matrix and are responsible for grain boundary pinning during forging. Here, orientation mapping is applied to study texture in the two phases and the nature of *γ*-*η* interphase relationships.

**Figure 11.**
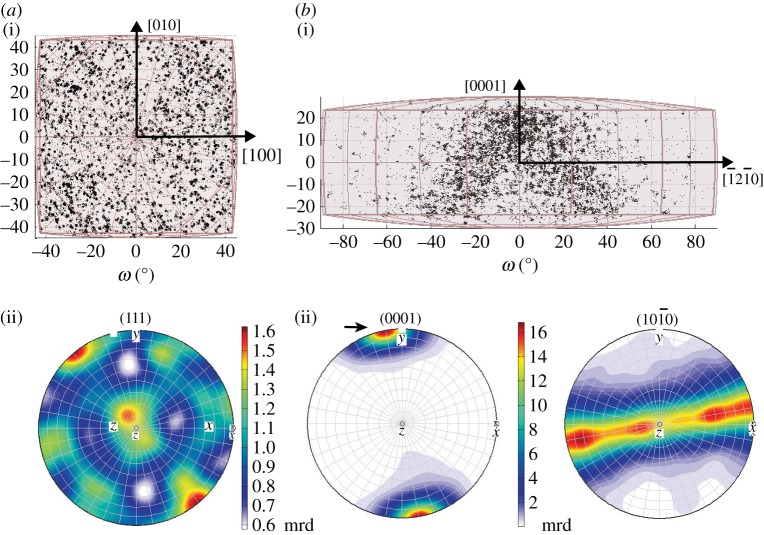
Fundamental zones for orientations of (*a*) *γ* phase (domain *c*) and (*b*) *η* phase (domain *f*). Pole figures are shown for comparison below the corresponding fundamental zone, plotted as orientation density function. Both representations can be used to identify the strong texture displayed by the *η* phase.

Texture is assessed by considering the deviation of an orientation distribution away from a random distribution [3]. Visualization of this distribution may be achieved by plotting the orientations of the phases in the appropriate fundamental zone of orientation space, as shown in figure 11. In the case of the cubic matrix (figure 11*a*), there is no significant clustering of the data within the fundamental zone, whereas, for the hexagonal precipitates (figure 11*b*), there is a strong texture resulting in two fibres in the orientation data. The same information is more conventionally presented in the form of pole figures (figure 11*a*(ii),*b*(ii)). The majority of *η*-phase particles have a similarly oriented (0001) pole with less strong preference for rotation about that pole. The 3D orientation space approach provides a means to visualize these aspects of texture in a single plot rather than requiring two pole figures. The finding of texture for *η* phase is consistent with alignment due to the flow stresses acting on *η* precipitates during the forging process and is not connected to a growth mechanism [82].

**Figure 12.**
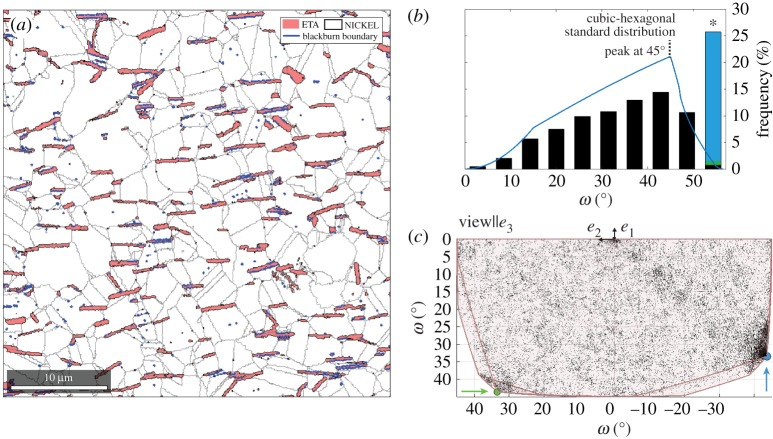
(*a*) Phase map with grain boundaries in black and interphase boundaries with Blackburn OR in blue, (*b*) misorientation angle distribution (1D) indicating high density at angles greater than 50°; *max. angle is 56.60°. For comparison: random distribution in blue. (*c*) 3D misorientation distribution of *γ*-*η* phase boundary in the appropriate fundamental zone (*a*/4), indicating that both, Blackburn and Crawley OR contribute to the high frequency at the max. angle.

Misorientations are commonly assessed using an angular distribution as displayed in figure 12*b* in the form of a histogram for *γ*-*η* boundary misorientations corresponding to the phase boundary line in (a). For comparison, a random distribution of misorientations for *m*$\bar{3}$*m* combined with 6/*mmm* symmetry is plotted as blue line with the maximum angle being 56.60° (marked with an *). The distribution of *γ*-*η* misorientations approximately follows the random distribution with a first peak at about 45°. Significant though is the peak at the maximum angle. In this plot alone, the origin of the peak cannot be determined.

However, plotting misorientations across *γ*-*η* phase boundaries in the fundamental zone for *m*$\bar{3}$*m* and 6/*mmm* point group symmetries, as shown in figure 12*c*, has some advantages. In this representation, two misorientation clusters around particular vertices indicated by blue and green circles can be identified. One of these (blue) is consistent with the Blackburn orientation relationship, which has been reported previously for ATI718Plus [83], while the second (green) is consistent with the Crawley orientation relationship, previously unreported for Nickel alloys, as follows:

$$\begin{array}{r} \begin{array}{rl} & \mathrm{Blackburn}\;{\left(11\bar{1}\right)}_{\gamma }∥{\left(0001\right)}_{\eta },\;{\left[1\bar{1}0\right]}_{\gamma }∥{\left[11\bar{2}0\right]}_{\eta }\\ & \mathrm{Crawley}\;\;\;{\left(11\bar{1}\right)}_{\gamma }∥{\left(0001\right)}_{\eta },\;{\left[1\bar{1}0\right]}_{\gamma }∥{\left[10\bar{1}0\right]}_{\eta }. \end{array} \end{array}$$

A more quantitative analysis of the misorientation distribution can be performed by isolating the misorientations near to a specified orientation relationship. Here, the misorientations near to the Blackburn orientation relationship are considered first. This result addresses the question if the aligned *η* particles may have lost their original orientation relationship with the surrounding matrix after forging and recrystallization. A homogeneous distribution would contain 0.2% of the data up to a distance of 3°, whereas in this case 17.7% of the data are contained in the angular range. The spatial distribution of the boundaries displaying the Blackburn OR was then considered plotting these boundaries in blue and it can be seen that several of the aligned *η*-precipitates adopt the Blackburn OR. Secondly, the same analysis is performed for the Crawley OR and one can find that 0.4% of the data are contained within a radius of 3° around the Crawley OR. Clearly, the preference for this configuration is less strong than for the Blackburn OR. Perhaps, a larger dataset than the 1304 *η* particles analysed here is needed to claim any physical impact.

This case study demonstrates the identification of orientation relationships in the *γ*-*η* system based on the observation of clusters in the 3D misorientation space. These clustered misorientations were then explored further with the statistical assessment of misorientation occurrence for the Blackburn and Crawley orientation relationships allowing physical insight into the continued relationship between the two phases. The spatial distribution of boundary misorientations was also used as a means to cross-check the occurrence and physical significance of clusters. Furthermore, the visualization of texture in 3D orientation space for *η* phase orientations, allowing individual grains to be identified, was illustrated.

### (d) Twinning and symplectite structures in anorthosites

Anorthosites are igneous rocks consisting of more than 80 mol-% of plagioclase, which are commonly found in *mafic* (magnesium-/iron-rich), layered intrusions. The samples studied here come from the largest such intrusion on Earth, the Bushveld Complex, South Africa. Plagioclase is one of the most common rock forming mineral series comprising solid solutions of the Albite–Anorthite series and compositions in layered intrusions can vary from An_78_ to An_45_ [84]. Here, twinning within the plagioclase component, taken as triclinic anorthite ($\bar{1}$, space group 2) and the misorientations between the plagioclase and intergrown augite, monoclinic (2/*m*, space group 19), in a *Symplectite* texture are revealed. Twins in plagioclase feldspars are commonly Albite and Pericline but a number of twinning relationships have been identified, as detailed in table 5 [85].

**Table 5.** Twin laws in plagioclase feldspar (anorthite) [86,85]; The composition plane is the plane of contact between the two parts of the twin. *Line colour in figure 13. Normal twins are considered type I and parallel type II. *Δ*M is the misorientation error between the cluster centre and the idealized notation.

name	misorientation axis/angle	twin type	composition plane	colour*	*Δ*M
X-law	⊥ (100) 180°	normal	(100)	green	3.0°
Albite	⊥ (010) 180°	normal	(010)	orange	1.2°
Manebach	⊥ (001) 180°	normal	(001)	blue	0.2°
Ala	[100] 180°	parallel	(0*kl*)	red	1.2°
Pericline	[010] 180°	parallel	(*h*0*l*)	cyan	1.0°
Carlsbad	[001] 180°	parallel	(*hk*0)	pink	0.1°

**Figure 13.**
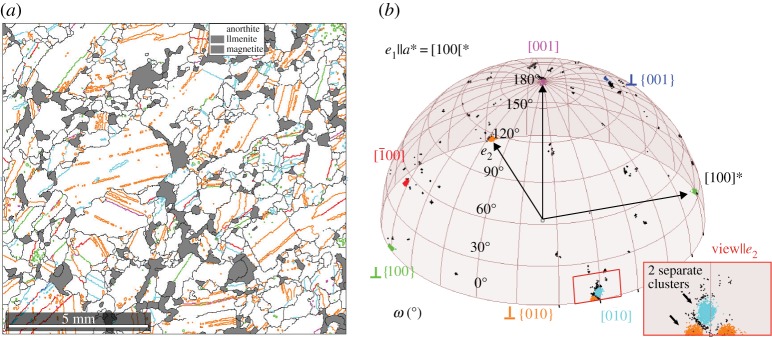
(*a*) Phase map (anorthite = white, other phases grey), twin boundaries are colour-coded (cf. table 5) (*b*) Selected anorthite boundary misorientations (*ω*>178°) in fundamental zone (*o*/2, cf. table 1).

Anorthite–anorthite misorientations were plotted spatially in a phase map and in the appropriate fundamental zone (*o*/2) as shown in figure 13. Clusters were identified near to the bounding hemisphere corresponding to twinning relationships, which all have misorientation angles of 180° in this system. Indeed, 48.2% of the data were found to be within 2° of the bounding hemisphere. Therefore, only those misorientations are considered here and plotted in figure 13*b*. Clusters with misorientation axes near ⊥{010} and [0$\bar{1}$0] (orange and cyan) correspond to the Albite and Pericline twin variants. These twin relationships are closely located in the misorientation space (inlay with details) and indeed 38.8% of the data are within 5° of these two relationships. The inlay shows proof that these two clusters are separate though. Further, clusters corresponding to: ⊥{100} - X-law, [$\bar{1}$00]—Ala, ⊥{001}—Manebach, and [001] Carlsbad twins were also identified with cluster centres of the last two closest to the idealized description (small *Δ*M).

The prevalence of the observed twin types was then calculated to be: Albite 54%, Pericline 24%, X-law 13%, Carlsbad 5%, Ala 2% and Manebach 1%; which is in agreement with work the done by Suwa [87] on another Bushveld sample, although no occurrence of Albite–Carlsbad twins was found in this study. The spatial occurrence of the twinning relationships was visualized by colouring boundaries according to their type, as shown. Most of the highlighted twin boundaries are straight continuous lines. However, a few appear rather dotted, for example, the green and cyan lines. They are also considered twin boundaries belonging to twins that could not be resolved with the chosen step size.

**Figure 14.**
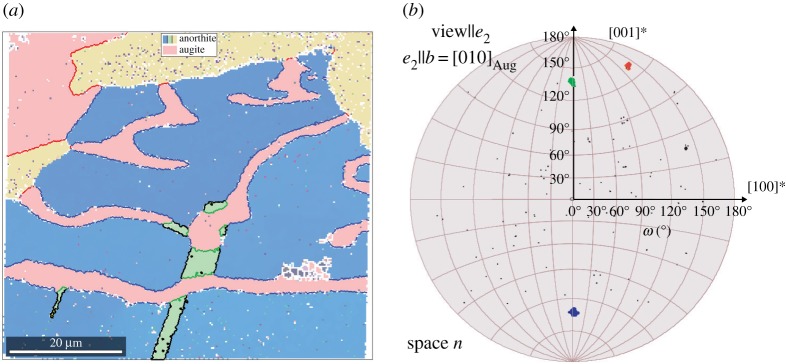
(*a*) Orientation map showing the occurrence of three orientations for anorthite (yellow, green and blue) and one for augite (pink) with the respective phase boundaries coloured red, green and blue. (*b*) Augite–anorthite misorientations in the appropriate fundamental zone (domain *n*); misorientation clusters coloured in accordance with phase boundaries in (*a*).

A second sample containing a two-phase intergrowth (Symplectite structure) between plagioclase and augite ((Ca, Mg, *Fe*2+, Al)_2_(Si, Al)_2_O_6_) was studied, as shown in figure 14. The augite phase adopts the same orientation (pink) in a grain (upper left corner) and in the vermicular structure intergrown with an anorthite grain (blue). The yellow anorthite grain on the top of the page is not part of the Symplectite fabric (microstructure) and twins (green) were observed in the anorthite. In the fundamental zone for augite–anorthite misorientations (figure 14*b*), three clusters are seen corresponding to the misorientations specific for each augite–anorthite pair in the dataset. The clusters in blue and green correspond to misorientations in the symplectite, the cluster in red does not. The blue cluster has a mean misorientation axis of [01$\bar{4}$] and angle 132° and occurs throughout the entire Symplectite region despite the vastly different curvature of the bounding plane due to the vermicular morphology of the augite. The green cluster has a misorientation axis [014] and angle 132°. The similarity of the axis-angle highlights their inter-relation and misorientation analysis shows that the green cluster corresponds to the Albite twin variant similar to the above example. Applying the Albite misorientation to the green cluster maps it onto the blue cluster, which suggests that the anorthite twin occurred after the Symplectite intergrowth had formed, perhaps a mechanical twin, which would be typical according to Suwa [87]. To understand the significance of the axis-angle pair (132° about [0$\bar{1}$4]), major poles for anorthite grains and augite were plotted, but no major poles coincided.

This case study demonstrates the identification of twin variants and orientation relationships of geological significance in crystal systems of relatively low symmetry (monoclinic/triclinic). This low symmetry results in large fundamental regions with bounds at 180°, which is also seen to be where important twinning information is localized. It is clear in this case that the popular Rodrigues–Frank representation would be useless as the boundary misorientations tend to be infinity in the important range. The large potential of this technique is the statistical analysis of occurring misorientations applied here to anorthite twinning. This information is characteristic for rock types as it helps analysing the formation of anorthite phase they contain and can be used to determine their provenance. That is e.g. to discern whether they are intrusive or extrusive rocks [88,89].

## 5. Summary and conclusion

This work demonstrates the utility of neo-Eulerian mappings (in this case the axis-angle parametrization) for gleaning insight from orientation and misorientation data through visualization in 3D vector spaces. A comprehensive overview of the most salient points of orientation mapping has also been provided with the aim of enabling experimentally oriented researchers to use and understand these methods. This is further facilitated through the recent rise of open source data analysis packages. In general, the salient features of the orientation data are indicated by clusters in the orientation space hinting that the development of specialized clustering algorithms would be valuable. A key feature of the analysis and workflow presented is also that correlations are drawn between the orientation space information and the real space spatial information, this is perhaps somewhat under-used in the literature and the methods set out make clear the value of this approach and provide a guide to the development of more automated analyses incorporating this principle. Overall, it is shown that these techniques provide an insightful representation of the data and allow a statistical assessment that goes beyond a mere visualization.

## Supplementary Material

Supplementary Material

## Supplementary Material

ESM files list

## Supplementary Material

9144317nkhzzqpxnqgc.zip

## Figures and Tables

**Figure 15.**
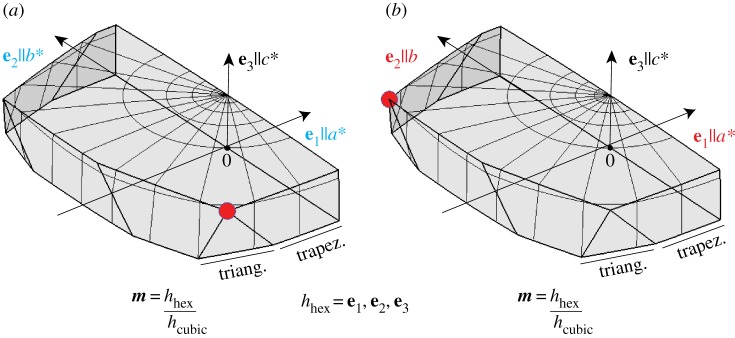
Influence of hexagonal reference frame on fundamental zone for cubic–hexagonal misorientations (*a*) first setting, cub: standard, hex: standard. Blackburn relation at lower corner (*b*) The second setting, cub: standard, hex: *e*_2_=**b**/*b*, *e*_3_=c*/c* leads to a rotation of the Blackburn relation to the upper corner, however, the domain is unchanged.

**Figure 16.**
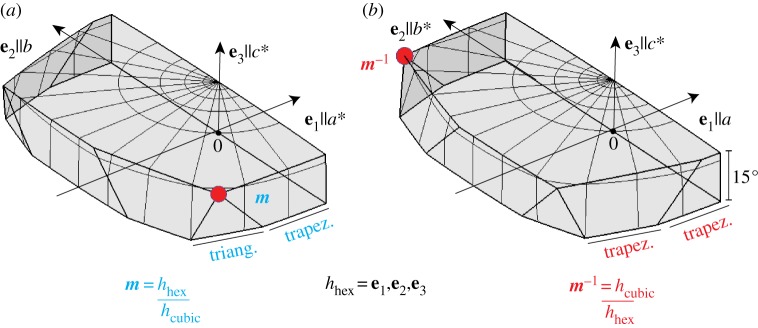
Influence of the sequence on the fundamental zone: Either (*a*) cubic–hexagonal misorientations (**e**_1_–**e**_3_ refer to cubic reference frame) or (*b*) hexagonal-cubic misorientations (**e**_1_–**e**_3_ refer to hexagonal reference frame) are formed. Note the change in geometry in (*b*) compared to (*a*) and the changed location of the Blackburn relation. Both FZ were formed with the standard definition for hexagonal symmetry as was done previously in figure 15*a*.

## Footnotes

1 Recently Morawiec has made a case for using crystallographic bases as an alternative [49], but this is not common at present.

2 This is consistent with the definition chosen in MTEX. However, it is inverse to the definition favoured by others [50].

3 In many texts, the specimen coordinate system is referred to in terms of the rolling direction, normal direction and transverse direction owing to a large number of studies interested in crystallographic texture induced by mechanical working.

4 The primary issues with plotting Euler angles are non-singularity of orientations, especially the identity, distorted volume and no direct reference with specimen axes [53–56].

5 It is noteworthy that some authors have applied a factor of 2 scaling to the rotational angle in the axis-angle representation in order to make the Taylor expansion of all neo-Eulerian mappings more similar at low angles. We have not adopted this convention here as we find the vector magnitude being directly the rotational angle to be convenient in a pragmatic sense.

6 The homochoric mapping is also related to the so-called cubochoric mapping in which efficient division of the space is possible [61].

7 Some authors [53] have suggested applying symmetry to the specimen coordinate system, but this can be confusing with respect to misorientations and is avoided in this work.

8 If the crystallographic point groups *S*_1_, *S*_2_ comprise *N*_1_ and *N*_2_ symmetry elements, then each misorientation generally has *N*_1_×*N*_2_ symmetry equivalents.

9 Specifically, if we denote *S*_*C*_=*S*_1_∩*S*_2_ the group of common symmetries of *S*_1_ and *S*_2_. Then for any misorientation *m* with rotational angle *ω*(*m*) and rotational axis ***ξ***(*m*), the symmetrically equivalent misorientations *s* *m* *s*^−1^, *s*∈*S*_*c*_ have the same rotational angle *ω*(*sms*^−1^)=*ω*(*m*) but different rotational axis ***ξ***(*sms*^−1^)=*s****ξ***(*m*).

10 The Greninger–Troiano OR, which is not further detailed here, leads to a value of 46.3%.

## Data accessibility

The data presented in this work are available at [48] along with matlab scripts to perform all analysis shown using the MTEX package.

## Appendix A. Computation of the fundamental zone

Fundamental zones were calculated following an approach similar to that of Morawiec [16] but making use of quaternion geometry rather than Rodrigues parameters.

Let us consider misorientations with respect to two point groups *S*_1_ and *S*_2_ and assume for the moment that *S*_1_ and *S*_2_ have no common symmetries, i.e. *S*_*C*_=*S*_1_∩*S*_2_={*id*}. In this case, the fundamental zone is completely characterized by the minimum misorientation angle, i.e. as set the set *F*_*S*_1_,*S*_2__ of misorientations ***m*** such that their rotational angle *ω*(***m***) is smaller than the misorientation angle *ω*(*s*_1_***m****s*_2_) of any symmetrically equivalent misorientation *s*_1_***m****s*_2_, *s*_1_∈*S*_1_, *s*_2_∈*S*_2_. Making use of the fact that the rotational angle *ω*(***m***) is the same as the angle *ω*(***m***,*id*) between the identity *id* and ***m*** the fundamental zone can be written as

$$\begin{array}{rl} F_{S_{1},S_{2}} & =\{\text{m}|\omega (\text{m})\leq \omega (s_{1}\text{m}s_{2}),\;s_{1}\in S_{1},\;s_{2}\in S_{2}\},\\ & =\{\text{m}|\omega (\text{m},\mathrm{id})\leq \omega (\text{m},s_{1}^{-1}s_{2}),\;s_{1}\in S_{1},\;s_{2}\in S_{2}\}. \end{array}$$

Consequently, the fundamental zone can be identified with the set of misorientations ***m*** that are closer to the identity than to any other misorientation *s*∈*S*=*S*_1_*S*_2_={*s*_1_*s*_2_|*s*_1_∈*S*_1_, *s*_2_∈*S*_2_} that is symmetrically equivalent to the identical misorientation *id*.

From a geometrical point of view, the above characterization of the fundamental zone is exactly the definition of a Voronoi cell. Hence, the fundamental zone can be computed by calculating the Voronoi tesselation of the rotation group with respect to the set of rotations *S*. To explain this another way, note that in terms of quaternions the misorientation space can be associated with the 3D sphere in the 4D Euclidean space, where antipodal points describe the same rotation. Since the angular distance between misorientations coincides with the angular distance of quaternions on the 3D sphere, the determination of the fundamental zone is nothing else than computing the Voronoi tessellation (tiling) of *S* on the 3D sphere.

Calculation of the Voronoi tessellation may be achieved by first computing the Delaunay triangulation [90] of *S* on the 3D sphere, e.g. using QHULL [91] which yields a list of tetrahedrons having the rotations in *S* as vertices. Next, we make use of the fact that the Delaunay triangulation is due to the Voronoi tessellation, i.e. the bounding faces of the fundamental region are just the bisectors of all edges of the Delaunay triangulation with one vertex being the identical misorientation.

In the case that *S*_1_ and *S*_2_ share common symmetries *S*_*C*_=*S*_1_∩*S*_2_≠{*id*}, the fundamental zone needs to restricted further. This is done by intersecting the Voronoi cell corresponding to *S*=*S*_1_*S*_2_ with the cone of axes defined by the IPF sector corresponding to the set of common symmetries *S*_*C*_.

## Appendix B. Conventions for combining symmetry groups

**(a) Aligning the crystal reference frame to crystal symmetry operators**

Orthonormal crystal reference frames that are introduced to simplify description of rotations can, in principle, be aligned arbitrarily with respect to the crystal structure. For consistency and to aid interpretation, conventions are adopted [52,92].

Two alignments are important: (i) Alignment of the symmetry operators with respect to the crystallographic axes (**a**, **b**, **c**) and (ii) alignment of the crystallographic axes (**a**, **b**, **c**) relative to the orthonormal axes *h*=(**e**_1_,**e**_2_,**e**_3_). Alignment of the symmetry operators with the crystallographic axes is set out systematically in the International Tables for Crystallography [93]. Here, the standard (first) settings were taken. To align crystallographic axes to orthonormal crystal reference axes, we set

A 1 $${\text{e}}_{1}=\frac{\text{a}}{a},\;{\text{e}}_{2}=\text{z}\times \text{x}\;\mathrm{and}\;{\text{e}}_{3}=\frac{{\text{c}}^{*}}{c^{*}},$$

where **e**_1_, **e**_2_, **e**_3_ are orthonormal basis vectors of the crystal reference frame *h* and **a, b, c** are crystallographic basis vectors.

Neither convention is consistently observed in the literature, sometimes for physical reasons and sometimes due to the limitations of indexation software. It is essential to note these alignments when drawing comparison between plots as the domain of the fundamental zones depends on them, as illustrated in figure 15, where changing the alignment of the orthonormal axes rotates the misorientations in the domain. In short, choose the basis consistently with the data you want to compare.

**(b) The order of symmetry operations**

Order matters for combining point groups *S*_1_ and *S*_2_. Symmetrically equivalent misorientations may be calculated as ***s***_2_ ***m*** ***s***_1_ or as ***s***_1_ ***m*** ***s***_2_ and if ***m*** belongs to the fundamental zone in the first case, then ***m***^−1^ belongs to the fundamental zone in the second. The former corresponds to *S*_1_ being taken as the basis reference frame and vice versa. The choice of basis reference frame has been approached following a ‘precipitation logic’ [29] and based on symmetry [30]. Precipitation logic suggests that when a precipitate forms in the matrix, the matrix reference frame will be the basis reference frame, whereas in the symmetry-based approach the highest symmetry system is taken as the basis reference frame. Here, the approach based on symmetry is favoured as it is more consistent. If both phases have the same symmetry, then precipitation logic may be applied.

Figure 16 illustrates the effect of reversing order in the combination of symmetry operations taking for example the so-called Blackburn orientation relationship [94]. This orientation relationship exists between certain cubic *γ*-Ti and hexagonal *α*-Ti phases according to

A 2 $${\left(11\bar{1}\right)}_{\gamma }∥{\left(0001\right)}_{\alpha },{\left[1\bar{1}0\right]}_{\gamma }∥{\left[11\bar{2}0\right]}_{\alpha }.$$

Depending on whether the *γ*-Ti or *α*-Ti phase is rotated onto the other to produce the Blackburn orientation relationship, either the misorientation ***m*** or its inverse ***m***^−1^ is formed. These misorientations are indicated by red dots in figure 16*a*,*b* and correspond to misorientations ***m*** of 56.6° about ***ξ***=[−0.7693, −0.5903, 0.2445] and ***m***^−1^ about ***ξ***=[−0.7693, 0.5903, 0.2445] respectively.^11^ This argument extends to all points of the fundamental zone and the resulting fundamental zones are related by inversion so cannot be rotated into coincidence.

## Appendix C. Combinations of all point groups

The correspondence between proper point groups and Laue groups required to select the correct fundamental zone in which to plot the data is given in table 6.

**Table 6.** The proper crystallographic point groups and corresponding Laue groups for selecting the correct fundamental zone in which to plot the data.

proper PG	432	23	622	6	422	4	32	3	222	2	1
Laue group	$m\bar{3}m$	$m\bar{3}$	6/*mmm*	6/*m*	4/*mmm*	4/*m*	$\bar{3}m$	$\bar{3}$	*mmm*	2/*m*	$\bar{1}$
