# Supplementary material for: On three-dimensional misorientation spaces
Source: Proc Math Phys Eng Sci. 2017 Oct 25;473(2206):20170274. doi: 10.1098/rspa.2017.0274 (PMC5666230; doi:10.1098/rspa.2017.0274)
Supplement: Supplementary Material [file rspa20170274supp1.pdf]

On Three-Dimensional Misorientation Spaces: Supplementary Material

Robert Krakow[1], Robbie J. Bennett[1], Duncan N. Johnstone[1], Zoja Vukmanovic[2], Wilberth Solano-Alvarez[1], Steven J. Lainé[1], Joshua F. Einsle[1,2], Paul A. Midgley[1], Catherine M.F. Rae[1], Ralf Hielscher[3]

[1]*Department of Materials Science and Metallurgy, University of Cambridge, 27 Charles Babbage Road, Cambridge, CB3 0FS, UK*
[2]*Department of Earth Sciences, University of Cambridge, Downing Street, Cambridge, CB2 3EQ, UK*
[3]*Applied Functional Analysis, TU Chemnitz, Germany*

Abstract

Determining the local orientation of crystals in engineering and geological materials has become routine with the advent of modern crystallographic mapping techniques. These techniques enable many thousands of orientation measurements to be made, directing attention towards how such data are best analysed and plotted. Here, we provide a guide to the visualisation of misorientation data in 3D vector spaces, reduced by crystal symmetry, to reveal crystallographic orientation relationships. By choosing an appropriate vector space, domains for all point group symmetries become accessible and fundamental zones are presented for combinations of all Laue classes. An analysis methodology is then developed and applied to identify crystallographic relationships in examples from materials science and geology. These examples highlight key advantages of analysing misorientations as 3D vectors. In particular, identification of misorientation clusters highlights the presence of previously unknown orientation relationships and helps determining active deformation mechanisms. Evaluation of cluster spread and centre allows for a more accurate description of transformation processes and quantification of clusters can be used for arguments of a sample's provenance.

Keywords: Misorientations, Orientation Relationships, Crystallography, Diffraction, EBSD, Steels

---

1. Materials & Methods

The methods of sample preparation and details of the acquisition of electron backscatter diffraction (EBSD) data for the examples presented in this work are detailed here alongside additional background of the samples studied.

1.1. Nanostructured Bainitic Steel

In this study, a nanostructured bainitic steel produced by TATA Steel UK was analysed using EBSD after extended rolling contact fatigue at room temperature. The experimental procedure has been previously published [1]. Figure 7 in the main text shows the ferrite and austenite distribution within a sample post deformation.

Bainite is a microstructure in steels that forms by the displace transformation of austenite at temperatures above which martensite starts to form (M_s), but below that (T_0) where austenite and ferrite of the same composition have the same free energy [2]. Such transformation is accompanied by a shape deformation, which is an invariant-plane strain with a large shear component. Three types of bainite can form: upper bainite, which is a non-lamellar aggregate of plate-shaped ferrite whose boundaries are decorated by elongated cementite, lower bainite, which has thinner interplatelet cementite but also fine carbide precipitates inside the ferrite, and carbide-free bainite where an addition of at least 1.5 wt% silicon restricts the precipitation of carbides due to the low solubility of this element in cementite. In all cases, bainite plates nucleate at the austenite grain boundaries and grow without diffusion creating plastic deformation of the austenite, which limits the length of the plates. Excess carbon is then partitioned into the residual austenite making the next ferrite plate grow from a carbon-enriched austenite. The process stops at the T_0 curve boundary, obtained by plotting the locus of the T_0 temperature against carbon concentration, where diffusionless transformation becomes impossible even if the austenite has not reached its equilibrium composition. This growing mechanism is known as the incomplete reaction phenomenon and explains the presence of carbon-saturated retained austenite in the product microstructure [3].

Nanostructured bainitic steel is a type of carbide-free bainite at a refined scale, making it in fact the world's first bulk nanostructured metal [4, 5]. Due to its high hardness (600-670 HV), strength (2-2.5 GPa), toughness (30-40 $\mathrm{MPa\,m^{1/2}}$) [6], and optimal resistance to ballistic impact [7], abrasive wear [8], rolling-sliding wear [9, 10, 11], fatigue [12, 13], and hydrogen embrittlement [14], it is currently produced as armour, with prospective uses as jet turbine shafts [15] and roller bearing components [16, 1]. These properties are achieved through a unique

combination of chemical composition and heat treatment that create a structure of fine bainitic ferrite platelets dispersed in a matrix of carbon-enriched austenite without carbide precipitation [17]. Besides the fine grain size, the toughness originates from the addition of 1.5-2 wt% silicon that suppresses cementite precipitation during the bainitic reaction, whereas the stabilised enriched austenite with no ductile-brittle transition temperature allows work hardening through its stress-induced transformation to martensite.

The nanostructured bainitic steel samples used in this study were tested under rolling contact fatigue and then sectioned along the radial cross section, mounted in bakelite, ground, polished, and etched in 2% nital as described in [16]. EBSD was then performed using an FEI Nova NanoSEM equipped with a field emission gun and a Bruker EBSD Detector operated at 20 kV with a working distance of 1.5 mm and a 0.04 mm aperture. EBDS data was acquired with a step size of 100 nm and the sample tilted to 70°.

1.2. Nickel-Base Superalloy

A sample of sub-solvus forged ATI718Plus ® was studied. The material was produced by ATI Speciality Materials via triple vacuum melting (vacuum induction melting/vacuum arc remelting/electro-slag remelting) and conversion from ingot to billet. This billet was then upset and subsolvus forged in a closed die by Otto Fuchs KG with subsequent standard heat treatment. Specimens were extracted from the forged product by Henschel KG using electrical discharge machining. Specimens were prepared for EBSD by grinding to 10 micron grit size followed by mechanical polishing using 1 micron diamond suspension and subsequently OPS colloidal silica.

EBSD was performed using an FEI Nova NanoSEM 450 equipped with a field emission gun and Bruker e^{-}Flash 1000 EBSD Detector operated at 20 kV with a working distance of 1.5 mm and a 40 micron aperture. EBDS data was acquired with a step size of 100 nm and the sample tilted to 70°.

1.3. Plagioclase Feldspar of the Bushveld Complex

Anorthosites are intrusive igneous rocks consisting of more than 80 mol-% of Plagioclase, which is one of the most common rock forming mineral series comprising solid solutions of predominantly Albite (Ab; $NaAlSi_3O_8$) and anorthite (An; $CaAlSi_3O_8$) with minor contributions of Orthoclase (Or; $KAlSi_3O_8$). Plagioclase compositions are often expressed in terms of the two predominant endmembers (Ab_xAn_{100-x}), or in terms of the anorthite mole fraction (i.e. (Ca/[Ca+Na+K]). Plagioclase composition in layered intrusions can vary from An_{78} to An_{45} [18].

Anorthosites are commonly found in mafic (magnesium/iron rich) layered intrusions and the two samples studied here come from the largest such intrusion on Earth, the Bushveld Complex, South Africa. The Bushveld Complex is a $\sim$ 6.5 km thick sequence of ultramafic and mafic rocks and was emplaced 2.06 Ga (billion years ago) [19] in several major magma recharged events [20]. The samples studied in here come from the Upper zone of the intrusion (100 m apart in stratigraphy), which hosts about 30 magnetite layers as well as world−class deposits of V, Ti and PGMs [21]. Here, EBSD is used to investigate twinning within the Plagioclase component as well as the orientation relationships between the Plagioclase and intergrown augite in a Symplectite texture.

The three phases present are, triclinic anorthite ($\bar{1}$, space group 2), trigonal ilmenite ($\bar{3}m1$, space group 164) and cubic magnetite ($m\bar{3}m$, space group 227). The lattice parameters and angles for anorthite structure were a=8.1732Å, b=12.8583Åand c=14.1703Å, as well as α=93.17°, β=115.95° and γ=91.22°. The symplectite fabric studied is a representative region of a larger sample shown in Figure 1.

2. References

[1] W. Solano-Alvarez, E. Pickering, M. Peet, K. Moore, J. Jaiswal, A. Bevan, H. Bhadeshia, Soft novel form of white-etching matter and ductile failure of carbide-free bainitic steels under rolling contact stresses, Acta Materialia 121 (2016) 215–226.

[2] H. Bhadeshia, Bainite in steels, Maney Publishing, London, third edition, 2015.

[3] H. Bhadeshia, Bainite in Steels, Maney Publishing, second edition, 2001.

[4] H. K. D. H. Bhadeshia, The first bulk nanostructured metal, Science and Technology of Advanced Materials 6996 (2013) 1–7.

[5] H. K. D. H. Bhadeshia, Nanostructured bainite, Proceedings of the Royal Society A: Mathematical, Physical and Engineering Sciences 466 (2010) 3–18.

[6] F. G. Caballero, H. K. D. H. Bhadeshia, Very strong bainite, Current Opinion in Solid State and Materials Science 8 (2004) 251–257.

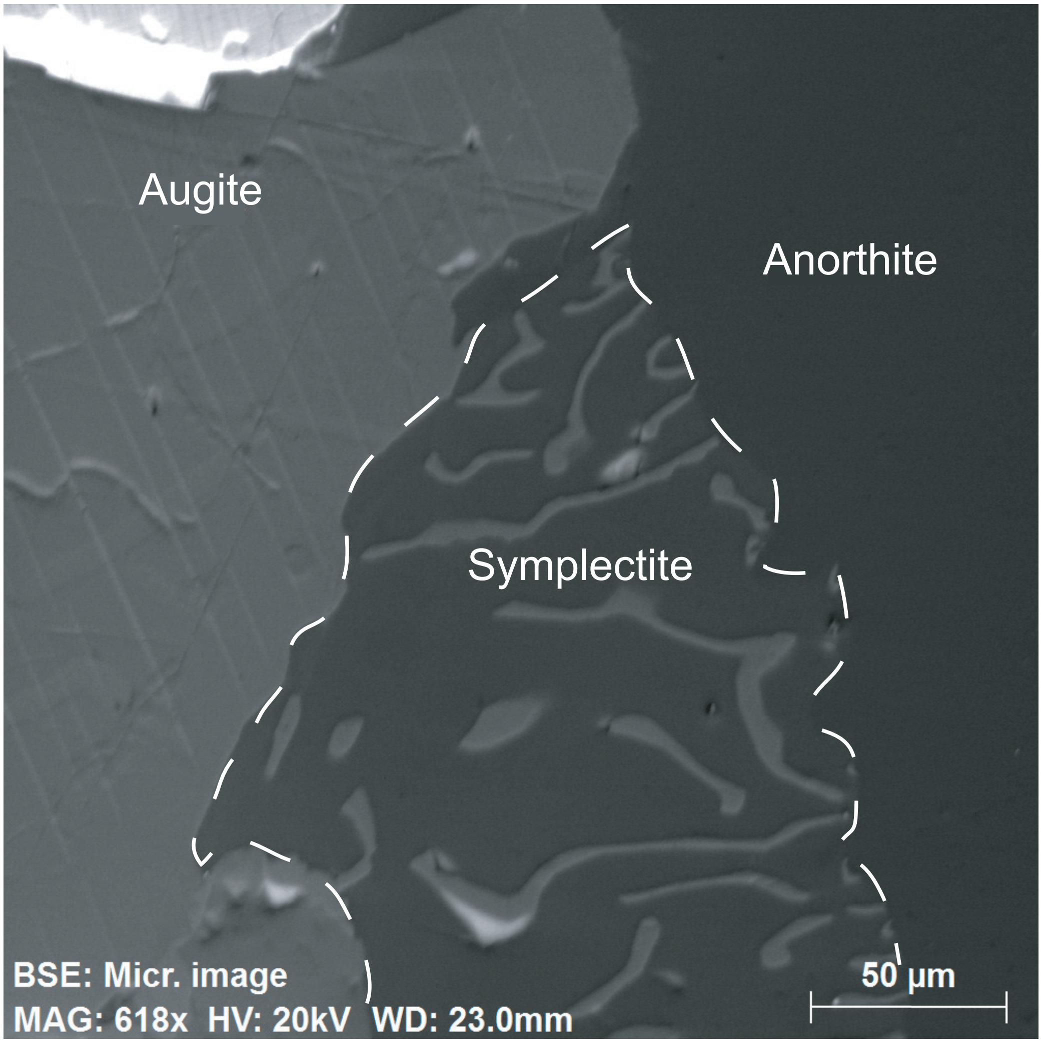

Figure 1: Backscattered electron image of Bushveld sample studied in case study 4 showing augite, anorthite and the intermediate Symplectite zones in the microstructure.

[7] L. C. D. Fielding, H. K. D. H. Bhadeshia, Shear band structure in ballistically tested bainitic steels, Materials Science and Technology 30 (2014) 812–817.

[8] S. Das Bakshi, P. H. Shipway, H. K. D. H. Bhadeshia, Three-body abrasive wear of fine pearlite, nanostructured bainite and martensite, Wear 308 (2013) 46–53.

[9] J. Yang, T. S. Wang, B. Zhang, F. C. Zhang, Sliding wear resistance and

worn surface microstructure of nanostructured bainitic steel, Wear 282-283 (2012) 81–84.

[10] A. Leiro, E. Vuorinen, K. G. Sundin, B. Prakash, T. Sourmail, V. Smanio, F. G. Caballero, C. Garcia-Mateo, R. Elvira, Wear of nano-structured carbide-free bainitic steels under dry rolling-sliding conditions, Wear 298-299 (2013) 42–47.

[11] S. Das Bakshi, A. Leiro, B. Prakash, H. K. D. H. Bhadeshia, Dry rolling/sliding wear of nanostructured bainite, Wear 316 (2014) 70–78.

[12] M. J. Peet, P. Hill, M. Rawson, S. Wood, H. K. D. H. Bhadeshia, Fatigue of extremely fine bainite, Materials Science and Technology 27 (2011) 119–123.

[13] T. Sourmail, F. Caballero, C. Garcia-Mateo, V. Smanio, C. Ziegler, M. Kuntz, R. Elvira, A. Leiro, E. Vuorinen, T. Teeri, Evaluation of potential of high Si high C steel nanostructured bainite for wear and fatigue applications, Materials Science and Technology (United Kingdom) 29 (2013) 1166–1173.

[14] L. C. D. Fielding, E. J. Song, D. K. Han, H. K. D. H. Bhadeshia, D.-W. Suh, Hydrogen diffusion and the percolation of austenite in nanostructured bainitic steel, Proceedings of the Royal Society A 470 (2014) 1–32.

[15] C. N. Hulme-Smith, I. Lonardelli, M. J. Peet, A. C. Dippel, H. K. D. H. Bhadeshia, Enhanced thermal stability in nanostructured bainitic steel, Scripta Materialia 69 (2013) 191–194.

[16] W. Solano-Alvarez, E. J. Pickering, H. K. D. H. Bhadeshia, Degradation of nanostructured bainitic steel under rolling contact fatigue, Materials Science and Engineering A 617 (2014) 156–164.

[17] F. G. Caballero, H. K. D. H. Bhadeshia, K. J. a. Mawella, D. G. Jones, P. Brown, Very strong low temperature bainite, Materials Science and Technology 18 (2002) 279–284.

[18] L. D. Ashwal, The temporality of anorthosites, The Canadian Mineralogist 48 (2010) 711–728.

[19] I. S. Buick, R. Maas, R. Gibson, Precise U-Pb titanite age constraints on the emplacement of the Bushveld Complex, South Africa, Journal of the Geological Society 158 (2001) 3–6.

[20] R. G. Cawthorn, F. Walraven, Emplacement and crystallization time for the Bushveld Complex, Journal of Petrology 39 (1998) 1669–1687.

[21] R. G. Cawthorn, S. Barnes, C. Ballhaus, K. N. Malitch, Platinum group element, chromium and vanadium deposits in mafic and ultramafic rocks, Economic Geology 100th Anni (2005) 215–249.
