# Supplementary material for: On three-dimensional misorientation spaces
Source: Proc Math Phys Eng Sci. 2017 Oct 25;473(2206):20170274. doi: 10.1098/rspa.2017.0274 (PMC5666230; doi:10.1098/rspa.2017.0274)
Supplement: ESM files list [file rspa20170274supp2.pdf]

“On Three-Dimensional Misorientation Spaces”

Electronic Supplementary Material

Robert Krakow[1], Robbie J. Bennett[1], Duncan N. Johnstone[1], Zoja Vukmanovic[2], Wilberth Solano-Alvarez[1], Steven J. Lainé[1], Joshua F. Einsle[1;2], Paul A. Midgley[1], Catherine M.F. Rae[1] and Ralf Hielscher[3]

[1] Department of Materials Science and Metallurgy, University of Cambridge, 27 Charles Babbage Road, Cambridge, CB3 0FS, UK

[2] Department of Earth Sciences, University of Cambridge, Downing Street, Cambridge, CB2 3EQ, UK

[3] Applied Functional Analysis, TU Chemnitz, Germany

List of ESM files

ESM file 1 (section3)	
Title	Matlab script
Legend	Running this Matlab script generates Figures in Section 3.

ESM file 2 (section 4a)	
Title	Matlab script generating Figures in Section 4a
Legend	Running this Matlab script generates Figures in Section 4a.

ESM file 3 (case_study_1_bainite_data)	
Title	CTF file containing data corresponding to ESM file 2
Legend	This file contains the raw EBSD data corresponding with ESM file 2, save in same repository.

ESM file 4 (section 4b)	
Title	Matlab script generating Figures in Section 4b
Legend	Running this Matlab script generates Figures in Section 4b.

ESM file 5 (case_study_2_titanium_data.crp)	
Title	CRP file containing data corresponding to ESM file 4
Legend	This file contains the raw EBSD data corresponding with ESM file 4, save in same repository.

ESM file 6 (case_study_2_titanium_data.crc)	
Title	CRC file containing data corresponding to ESM file 4
Legend	This file contains the raw EBSD data corresponding with ESM file 4, save in same repository.

ESM file 7 (section 4c)	
Title	Matlab script generating Figures in Section 4c
Legend	Running this Matlab script generates Figures in Section 4c.

ESM file 8 (case_study_3_superalloy_data)	
Title	CRP file containing data corresponding to ESM file 7
Legend	This file contains the raw EBSD data corresponding with ESM file 7, save in same repository.

ESM file 9 (section 4d1)	
Title	Matlab script generating Figures in Section 4d1
Legend	Running this Matlab script generates Figures in Section 4d1.

ESM file 10 (case_study_4a_anorthite_data)	
Title	CRP file containing data corresponding to ESM file 9
Legend	This file contains the raw EBSD data corresponding with ESM file 9, save in same repository.

ESM file 11 (section 4d2)	
Title	Matlab script generating Figures in Section 4d2
Legend	Running this Matlab script generates Figures in Section 4d2.

ESM file 12 (case_study_4b_symplectite_data)	
Title	CRP file containing data corresponding to ESM file 11
Legend	This file contains the raw EBSD data corresponding with ESM file 11, save in same repository.

Files can be downloaded from:

https://doi.org/10.17863/CAM.8815
