# Supplementary material for: On three-dimensional misorientation spaces
Source: Proc Math Phys Eng Sci. 2017 Oct 25;473(2206):20170274. doi: 10.1098/rspa.2017.0274 (PMC5666230; doi:10.1098/rspa.2017.0274)
Supplement: 9144317nkhzzqpxnqgc.zip [file rspa20170274supp3.zip › 9144317nkhzzqpxnqgc/Instructions.pdf]

# 1. Instructions for RS LaTeX Template

This latex class file is available for authors to prepare the manuscript for the Royal Society journals. It is assumed that the authors are familiar with either plain TeX, LaTeX, $\mathcal{AMS}$-TeX or a standard latex set-up, hence only the essential points are described in this document. For more details please see the LaTeX User's Guide or The not so short introduction to LaTeX $2_\varepsilon$.

# 2. Installation

Within the supplied set of files, `rsos.cls` need to be copied into a directory where tex looks for input files. The other files need to be kept as a reference while preparing your manuscript. Please use pre-defined commands from `Author_tex.tex` for title, authors, address, abstract, keywords, body etc.

# 3. How to start using rsos.cls

Before you type anything that actually appears in the paper you need to include a `\documentclass{rsos}` command at the very beginning and then, the two commands that have to be part of any latex document, `\begin{document}` at the start and the `\end{document}` at the end of your paper. The main structure of your document should be as follows:

```
\documentclass{rsos}
\begin{document}
\title{...}
\author{....}
\address{...}
\subject{...}
\keywords{...}
\corres{...}

\begin{abstract}
..........
..........
\end{abstract}

\begin{fmtext}
............
\end{fmtext}

\maketitle
....
\section{....}
...
\subsection{....}
....
\end{document}
```

Note: In order to insert few lines on first page use the command `\begin{fmtext}....\end{fmtext}` before `\maketitle` command. Please look into sample.tex for more clarification.

# 4. Preamble

In the preamble portion i.e. before `\begin{document}` insert the respective journal name: `\jname{rspa}` and `\Journal{Proc R Soc A\ }` to get journal name in running heads.

## 5. Packages used by the class file

There are some packages that are essential when using the class file:

`amsmath amssymb amsfonts amsthm graphicx endfloat endnotes setspace verbatim geometry times helvet courier mathtime bm url babel dcolumn`

Some commonly-used packages are already used by this class file:

`xspace amscd rotating latexsym multicol array algorithm subfigure`

Note: For the acknowledgement section use the command `\ack{}`.

The rsos file is intended as a guide only. Article lengths based on this estimation may be subject to change when the article is prepared for publication. Authors whose article is estimated close to the page charge limit should contact the office regarding page charges.
