# Supplementary material for: On three-dimensional misorientation spaces
Source: Proc Math Phys Eng Sci. 2017 Oct 25;473(2206):20170274. doi: 10.1098/rspa.2017.0274 (PMC5666230; doi:10.1098/rspa.2017.0274)
Supplement: 9144317nkhzzqpxnqgc.zip [file rspa20170274supp3.zip › 9144317nkhzzqpxnqgc/author_tex.pdf]

ROYAL SOCIETY OPEN SCIENCE

rsos.royalsocietypublishing.org

Research

Article submitted to journal

Subject Areas:

xxxxx, xxxxx, xxxx

Keywords:

xxxx, xxxx, xxxx

Author for correspondence:

Insert corresponding author name

e-mail: xxx@xxxx.xx.xx

THE ROYAL SOCIETY
PUBLISHING

Insert the article title here

X. X. First author[1], X. Second author[2] and X. Third author[3]

[1]First author address
[2]Second author address
[3]Third author address

The abstract text goes here. The abstract text goes here. The abstract text goes here. The abstract text goes here. The abstract text goes here. The abstract text goes here. The abstract text goes here. The abstract text goes here.

1. Insert A head here

This demo file is intended to serve as a "starter file" for rsproca journal papers produced under LaTeX using rsproca.cls v1.5e.

(a) Insert B head here

Subsection text here.

(i) Insert C head here

Subsubsection text here.

2. Equations

Sample equations.

$$\begin{aligned}\frac{\partial u(t,x)}{\partial t} &= Au(t,x)\left(1-\frac{u(t,x)}{K}\right)-B\frac{u(t-\tau,x)w(t,x)}{1+Eu(t-\tau,x)},\\ \frac{\partial w(t,x)}{\partial t} &= \delta\frac{\partial^2 w(t,x)}{\partial x^2}-Cw(t,x)+D\frac{u(t-\tau,x)w(t,x)}{1+Eu(t-\tau,x)},\end{aligned} \tag{2.1}$$

$$\begin{aligned}\frac{dU}{dt} &= \alpha U(t)(\gamma-U(t))-\frac{U(t-\tau)W(t)}{1+U(t-\tau)},\\ \frac{dW}{dt} &= -W(t)+\beta\frac{U(t-\tau)W(t)}{1+U(t-\tau)}.\end{aligned} \tag{2.2}$$

$$\begin{aligned}\frac{\partial(F_1,F_2)}{\partial(c,\omega)}_{(c_0,\omega_0)} &= \left|\begin{matrix}\frac{\partial F_1}{\partial c} & \frac{\partial F_1}{\partial \omega}\\ \frac{\partial F_2}{\partial c} & \frac{\partial F_2}{\partial \omega}\end{matrix}\right|_{(c_0,\omega_0)}\\ &= -4c_0q\omega_0-4c_0\omega_0p^2=-4c_0\omega_0(q+p^2)>0.\end{aligned} \tag{2.3}$$

© 2014 The Authors. Published by the Royal Society under the terms of the Creative Commons Attribution License http://creativecommons.org/licenses/by/4.0/, which permits unrestricted use, provided the original author and source are credited.

3. Enunciations

Theorem 3.1. *Assume that* $\alpha > 0, \gamma > 1, \beta > \frac{\gamma+1}{\gamma-1}$. *Then there exists a small* $\tau_1 > 0$, *such that for* $\tau \in [0, \tau_1)$, *if* c *crosses* $c(\tau)$ *from the direction of to a small amplitude periodic traveling wave solution of (2.1), and the period of* $(\check{u}^p(s), \check{w}^p(s))$ *is*

$$\check{T}(c) = c \cdot \left[\frac{2\pi}{\omega(\tau)} + O(c - c(\tau))\right].$$

Condition 3.1. *From (0.8) and (2.10), it holds* $\frac{d\omega}{d\tau} < 0, \frac{dc}{d\tau} < 0$ *for* $\tau \in [0, \tau_1)$. *This fact yields that the system (2.1) with delay* $\tau > 0$ *has the periodic traveling waves for smaller wave speed* c *than that the system (2.1) with* $\tau = 0$ *does. That is, the delay perturbation stimulates an early occurrence of the traveling waves.*

4. Figures & Tables

The output for figure is:

Figure 1. Insert figure caption here

The output for table is:

Table 1. An Example of a Table

date	Dutch policy	date	European policy
1988	Memorandum Prevention	1985	European Directive (85/339)
1991–1997	**Packaging Covenant I**		
1994	Law Environmental Management	1994	European Directive (94/62)
1997	Agreement Packaging and Packaging Waste		
1998–2002	**Packaging Covenant II**		
2003–2005	**Packaging Covenant III**		
2006–2007	**Decree on Packaging and paper**		

5. Conclusion

The conclusion text goes here.

Acknowledgment

Insert the Acknowledgment text here.

References

1. Allwood JM, Cullen JM. 2011 *Sustainable materials: with both eyes open*. Cambridge, UK: UIT Cambridge. See http://www.withbotheyesopen.com.
2. MacKay DJC. 2008 *Sustainable energy: without the hot air*. Cambridge, UK: UIT Cambridge. See http://www.withouthotair.com.
3. Gallman PG. 2011 *Green alternatives and national energy strategy: the facts behind the headlines*. Baltimore, MD: Johns Hopkins University Press.
4. MacKay DJC. 2013. Solar energy in the context of energy use, energy transportation, and energy storage. *Proc. R. Soc. A* **371**.
