# Supplementary figures and images for: On three-dimensional misorientation spaces
Source: Proc Math Phys Eng Sci. 2017 Oct 25;473(2206):20170274. doi: 10.1098/rspa.2017.0274 (PMC5666230; doi:10.1098/rspa.2017.0274)

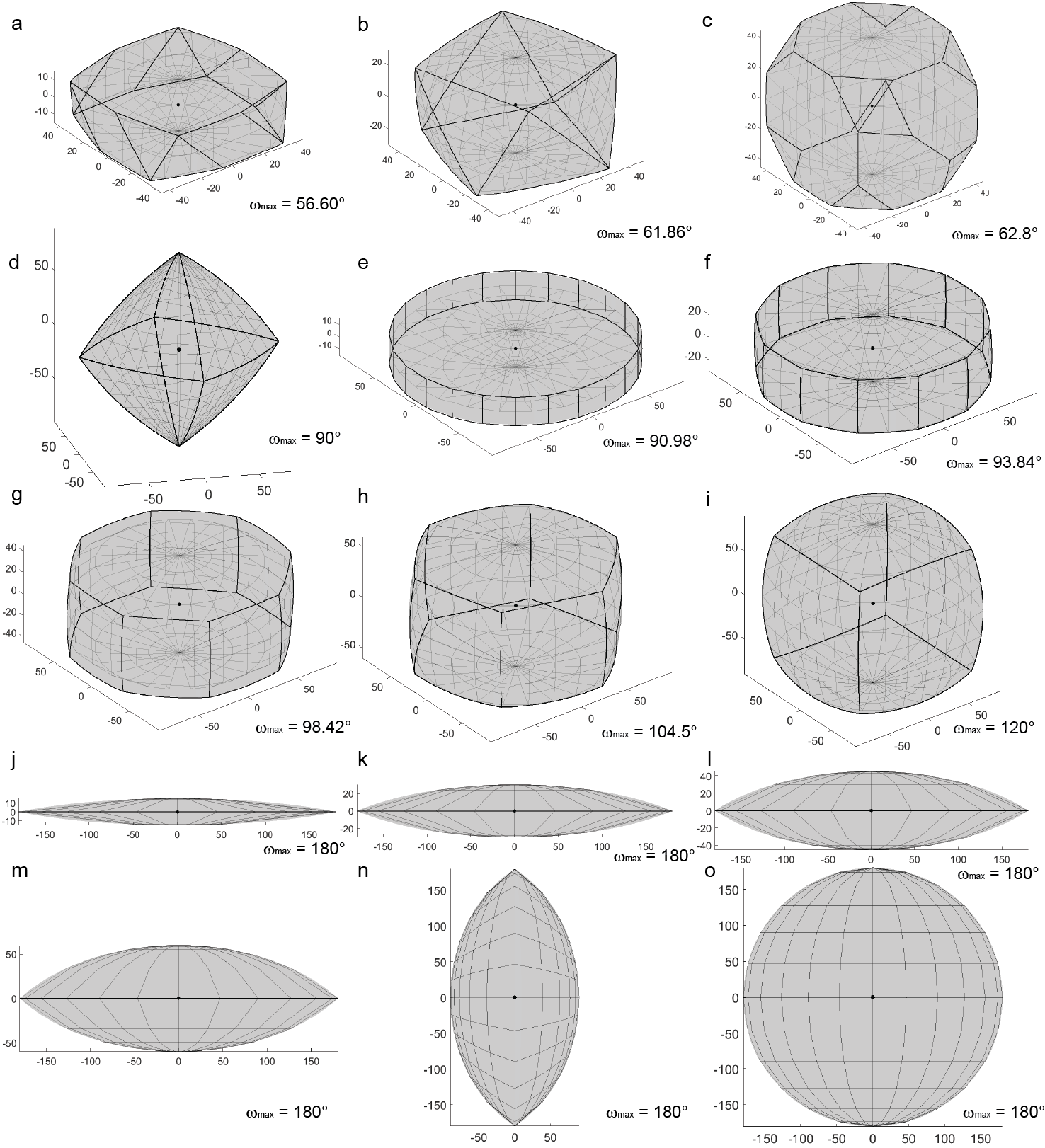

Supplement: 9144317nkhzzqpxnqgc.zip [file rspa20170274supp3.zip › 9144317nkhzzqpxnqgc/15_unique_spaces5.png]

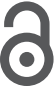

Supplement: 9144317nkhzzqpxnqgc.zip [file rspa20170274supp3.zip › 9144317nkhzzqpxnqgc/openaccesslogo_bw.pdf]

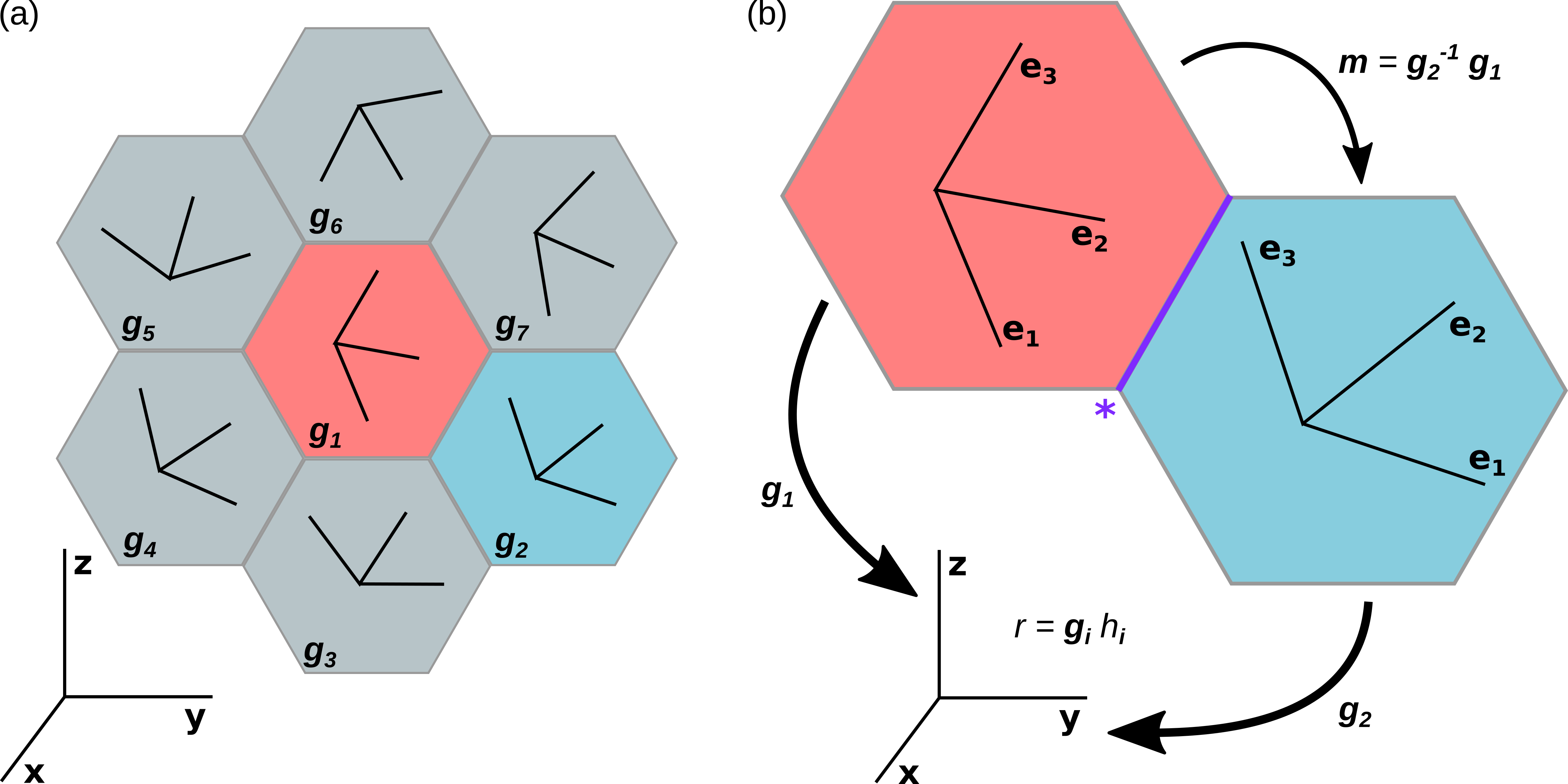

Supplement: 9144317nkhzzqpxnqgc.zip [file rspa20170274supp3.zip › 9144317nkhzzqpxnqgc/orientation_mapping.png]

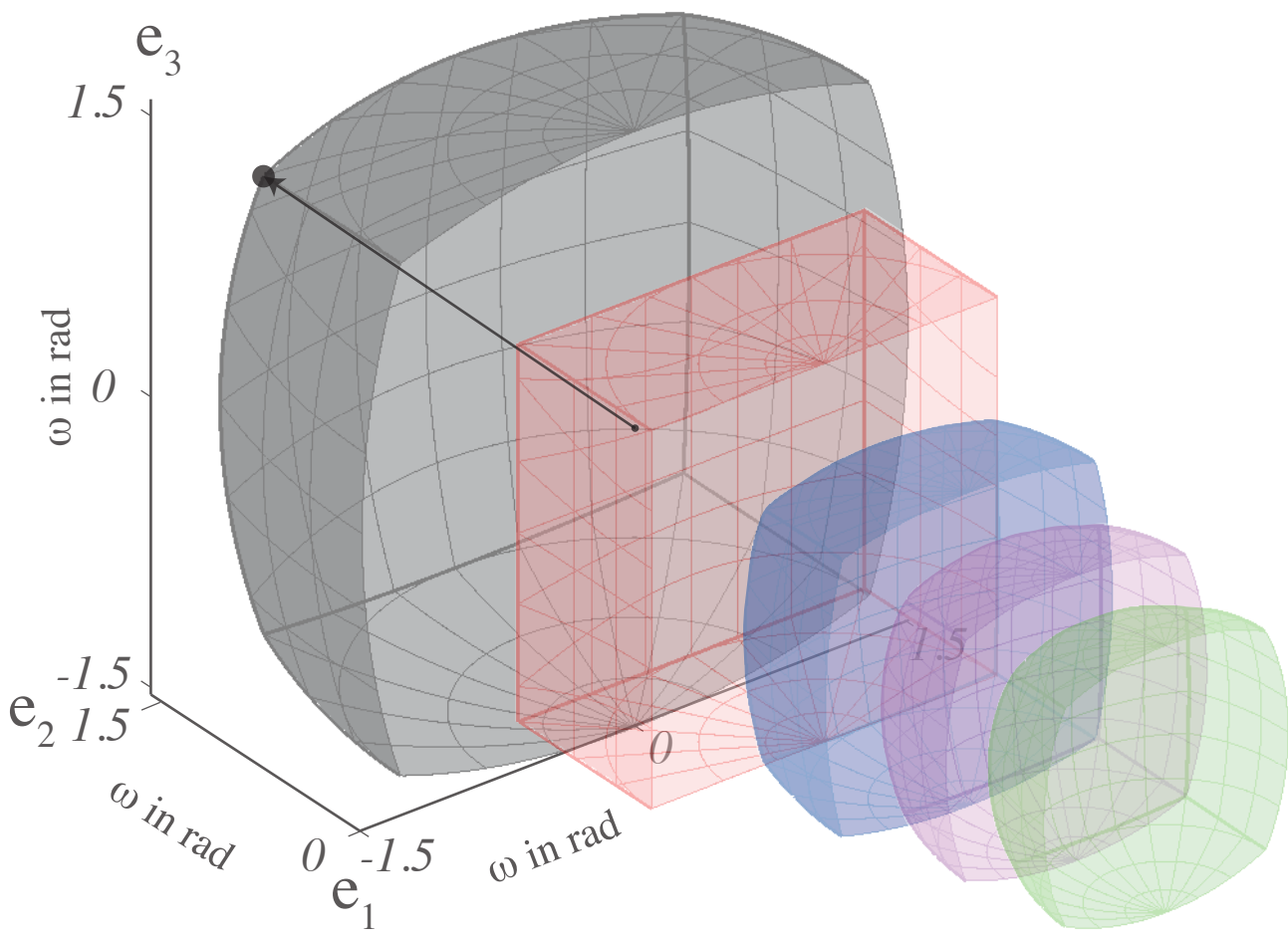

Supplement: 9144317nkhzzqpxnqgc.zip [file rspa20170274supp3.zip › 9144317nkhzzqpxnqgc/Vector_Constructions.pdf]

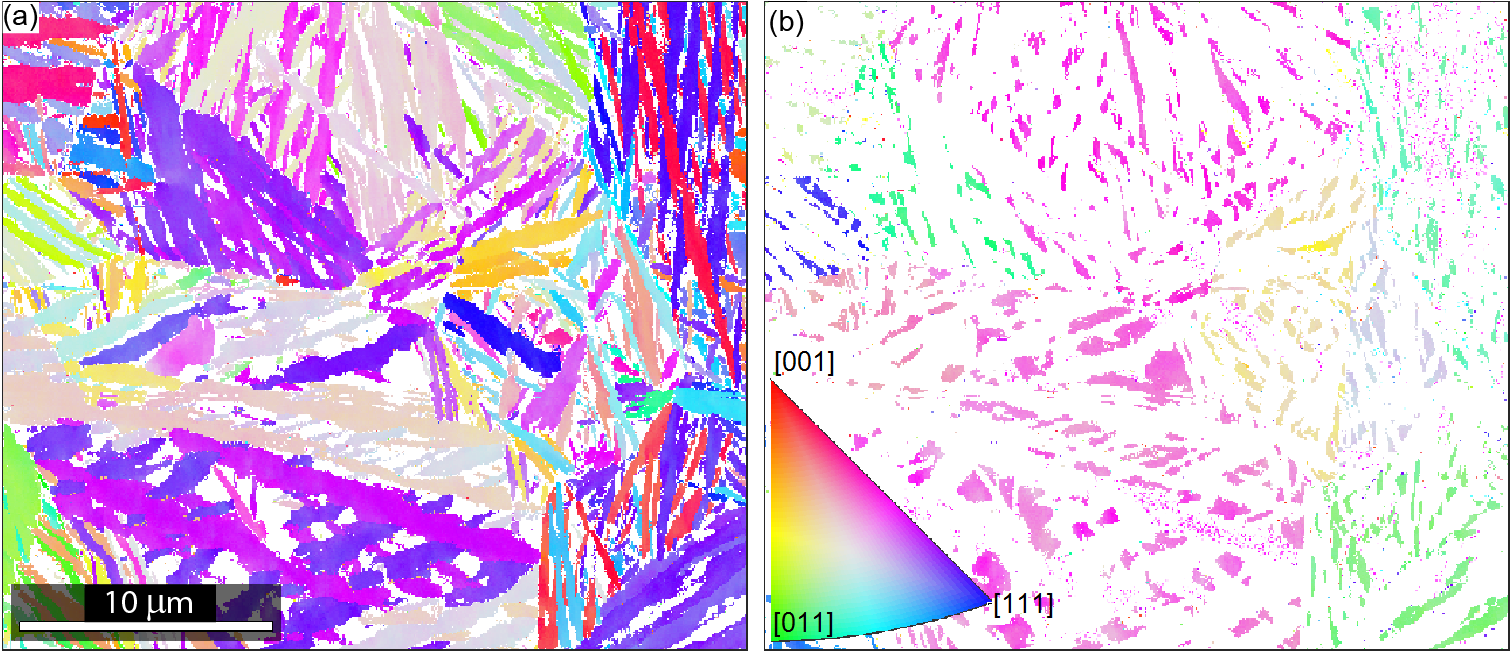

Supplement: 9144317nkhzzqpxnqgc.zip [file rspa20170274supp3.zip › 9144317nkhzzqpxnqgc/B_orientation_map_bainite2.png]

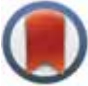

CrossMark

[click for updates](#)

Supplement: 9144317nkhzzqpxnqgc.zip [file rspa20170274supp3.zip › 9144317nkhzzqpxnqgc/RS_crossmark_logo.pdf]

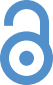

Supplement: 9144317nkhzzqpxnqgc.zip [file rspa20170274supp3.zip › 9144317nkhzzqpxnqgc/RSTA_OpenAccesslogo_RGB.pdf]
